# Familial Infertility (Azoospermia and Cryptozoospermia) in Two Brothers—Carriers of t(1;7) Complex Chromosomal Rearrangement (CCR):  Molecular Cytogenetic Analysis

**DOI:** 10.3390/ijms21124559

**Published:** 2020-06-26

**Authors:** Marta Olszewska, Tomasz Stokowy, Nijole Pollock, Nataliya Huleyuk, Andrew Georgiadis, Svetlana Yatsenko, Danuta Zastavna, Alexander N. Yatsenko, Maciej Kurpisz

**Affiliations:** 1 Institute of Human Genetics, Polish Academy of Sciences, Strzeszynska 32, 60-479 Poznan, Poland; marta.olszewska@igcz.poznan.pl; 2 Department of Clinical Science, University of Bergen, Postboks 7804, 5020 Bergen, Norway; tomasz.stokowy@k2.uib.no; 3 Department of OBGYN and Reproductive Science, Magee-Womens Research Institute, University of Pittsburgh, Pittsburgh, PA 15213, USA; pollocknc@mwri.magee.edu (N.P.); georgiadisa@mwri.magee.edu (A.G.); yatsenkosa@mail.magee.edu (S.Y.); yatsenkoan@mwri.magee.edu (A.N.Y.); 4 Institute of Hereditary Pathology, Ukrainian Academy of Medical Sciences, Lysenko Str. 31a, 79000 Lviv, Ukraine; huleyuk@yahoo.com (N.H.); zastavna.d@ihp.lviv.ua (D.Z.); 5 Department of Biotechnology and Bioinformatics, Faculty of Chemistry, Rzeszow University of Technology, Al. Powst. Warszawy 6, 35-959 Rzeszow, Poland

**Keywords:** azoospermia, cryptozoospermia, complex chromosomal rearrangement, chromosome translocation, sperm chromosomes, male infertility, male meiosis

## Abstract

Structural aberrations involving more than two breakpoints on two or more chromosomes are known as complex chromosomal rearrangements (CCRs). They can reduce fertility through gametogenesis arrest developed due to disrupted chromosomal pairing in the pachytene stage. We present a familial case of two infertile brothers (with azoospermia and cryptozoospermia) and their mother, carriers of an exceptional type of CCR involving chromosomes 1 and 7 and three breakpoints. The aim was to identify whether meiotic disruption was caused by CCR and/or genomic mutations. Additionally, we performed a literature survey for male CCR carriers with reproductive failures. The characterization of the CCR chromosomes and potential genomic aberrations was performed using: G-banding using trypsin and Giemsa staining (GTG banding), fluorescent in situ hybridization (FISH) (including multicolor FISH (mFISH) and bacterial artificial chromosome (BAC)-FISH), and genome-wide array comparative genomic hybridization (aCGH). The CCR description was established as: der(1)(1qter->1q42.3::1p21->1q42.3::7p14.3->7pter), der(7)(1pter->1p2 1::7p14.3->7qter). aCGH revealed three rare genes variants: *ASMT*, *GARNL3*, and *SESTD1*, which were ruled out due to unlikely biological functions. The aCGH analysis of three breakpoint CCR regions did not reveal copy number variations (CNVs) with biologically plausible genes. Synaptonemal complex evaluation (brother-1; spermatocytes II/oligobiopsy; the silver staining technique) showed incomplete conjugation of the chromosomes. Associations between CCR and the sex chromosomes (by FISH) were not found. A meiotic segregation pattern (brother-2; ejaculated spermatozoa; FISH) revealed 29.21% genetically normal/balanced spermatozoa. The aCGH analysis could not detect smaller intergenic CNVs of few kb or smaller (indels of single exons or few nucleotides). Since chromosomal aberrations frequently do not affect the phenotype of the carrier, in contrast to the negative influence on spermatogenesis, there is an obvious need for genomic sequencing to investigate the point mutations that may be responsible for the differences between the azoospermic and cryptozoospermic phenotypes observed in a family. Progeny from the same parents provide a unique opportunity to discover a novel genomic background of male infertility.

## 1. Introduction

Infertility affects approximately 10–18% of couples of reproductive age [1,2,3]. It is estimated that approximately 7% of men and 12% of women worldwide are affected and approximately 40–60% of all infertility cases originate from the male side [4,5]. There are multiple reasons for male infertility, including genetic factors, which determine approximately 10–15% of revealed diminished fertility cases; and approximately 25% of infertile males are diagnosed as idiopathic (unexplained etiology) [1,5,6]. Apart from gene mutations that are clearly linked to male infertility [6,7,8], chromosomal aberrations may also underlie male reproductive problems. The frequency of all chromosomal aberrations detected in the lymphocytes of fertile males is about 0.7%, while in infertile men this increases five-fold [9].

The most common detectable aberrations are aneuploidy (the presence of an additional chromosome) and structural aberrations of the chromosomes (reciprocal chromosome translocations—RCTs; and Robertsonian chromosome translocations—RobTs (about 1/500–1/700 live births)) [3,10]. In a population of infertile males, the probability of chromosome aberrations is several times higher when compared to the whole population (i.e., for RCT: 1% of infertile males are carriers, while in oligozoospermia, RCTs are approximately 16% of all identified aberrations; in azoospermia cases, this value is about 4%) [3]. Unknown genetic and epigenetic issues, hormonal abnormalities, varicocele, cryptorchidism, reactive oxygen species (ROS), and environmental factors (i.e., pollution, lifestyle, drugs, smoking, etc.) are listed as causes of male reproductive failure [11]. A lack of spermatozoa in semen (azoospermia) may arise either from the obstruction of the vas deferens (obstructive azoospermia, OA, with preserved spermatogenesis in the testis; 15–40% of azoospermic men) or as a consequence of a spermatogenetic arrest (nonobstructive azoospermia, NOA), which appears in almost 1% of the total male population and increases to 15–20% of infertile males [2,12]. The frequency of chromosomal aberrations in males with azoospermia rises to 20% [2].

Structurally balanced or unbalanced aberrations involving more than two breakpoints on two or more chromosomes, known as complex chromosomal rearrangements (CCRs), have been described in over 250 papers [13,14,15]. Approximately 75% of CCRs appear *de novo* or are inherited maternally (70% of all familial cases) [16,17]. There are four types of CCR according to the combination of the number of chromosomes and breakpoints involved. Types I–III are genetically balanced, while type IV is related to the presence of indels or the disruption of a gene [17,18,19,20]. Type I is the simplest and is characterized by an equal number of chromosomes and breakpoints involved in the CCR. This is called a “three-way rearrangement” (three chromosomes with one breakpoint per chromosome), and is the most often observed among all CCR cases and is inherited maternally. Type II, the so called “exceptional complex chromosomal rearrangement,” mainly appears *de novo* and is characterized by at least two breakpoints on the one of the chromosomes involved (i.e., reciprocal chromosomal translocation RCT, where one of the chromosomes consists of an inversion or insertion). The maximum number of chromosomes involved in type II is seven with a total number of 15 breakpoints [21]. Type III, also called the “double/triple two-way translocation,” is the coincidental presence of two or three separate reciprocal or Robertsonian chromosome translocations [17]. Type IV, which is genetically imbalanced, and also called an “insertional translocation,” is formed as a result of at least three breakpoints that lead to a variable number of copies of a DNA fragment [19].

CCR cases typically involve three (30%) or four (29%) chromosomal breakpoints. A higher number of breakpoints determines a higher risk of phenotypic abnormalities in a carrier (30–50%) or their offspring (20–90%) [13,14,20,21,22,23,24]. The high risk of abnormalities is estimated as 3.5% per breakpoint in CCR [14]. Drastic fertility reduction has been observed in CCR carriers, mostly from gametogenesis arrest, as a result of the disrupted pairings of chromosomes during the meiotic pachytene stage [13,24,25]. This is linked to the more complex character of the rearrangement, which involves more than two chromosomes, or a number of breakpoints that is higher than the number of chromosomes involved in the CCR. Chromosome pairing is linked to meiotic multivalent formation and possible impairment of the chromosome ends. Such impaired ends allow the conjugation of other chromosomes, mainly sex chromosomes, leading to diminished spermatogenesis manifested in decreased semen parameters; i.e., azoospermia (spermatogenetic block) or oligozoospermia (the elimination of genetically unbalanced gametes by molecular mechanisms) [26,27].

Additionally, some chromosomal fragments may stay asynapsed, leading to developmental block of spermatocytes. Alike simple RCTs, in CCRs, the behavior of multivalent formation also depends on the type of chromosomes involved (autosomes or gonosomes, acrocentrics). The chromosomal features describing the multivalent geometry (the sizes of interstitial and translocated fragments, the distances of the breakpoints to the centromeres, and the presence and number of interstitial chiasmata) determine the pathways of chromosomal disjunction [27,28]. Currently, only 161 balanced CCR male carriers have been described, and an evaluation of the reproductive status was performed in only 64 cases (summarized in Supplementary Table S1).

Researchers found that sperm cells of approximately 50% of genetically balanced male CCR carriers were able to fertilize (Supplementary Table S1) [14]. In several cases, the meiotic segregation of chromosomes involved in the rearrangement was examined, with the frequency of genetically unbalanced spermatozoa ranging from 69% to 88%. Such high values arise from the complexity of the rearrangement: i.e., simple type I CCR with three chromosomes involved, each with one breakpoint, generates 64 various unbalanced genotypes in spermatozoa, while in simple RCTs, a quadrivalent formation generates 16 different types of meiotic segregants. This fact clearly indicates an increased risk of reproductive failures in CCR carriers, mostly leading to miscarriages (Supplementary Table S1) [15,29].

Here, we present a familial case of two infertile brothers (azoospermic and cryptozoospermic) and their mother, all carriers of CCR involving chromosomes 1 and 7 and three breakpoints, resulting in the so-called exceptional type of CCR (type II). The purpose of the study was to identify whether meiotic disruption was caused by chromosomal rearrangement and/or by genomic mutations. Additionally, we performed a literature survey for male CCR carriers described according to their reproductive failures.

## 2. Results

**Figure 1:**
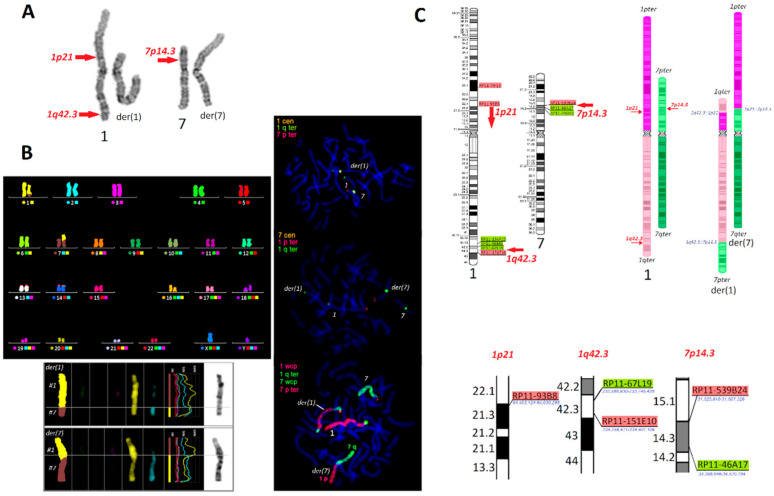
The characteristics of the chromosomes involved in complex chromosomal rearrangements (CCRs) t(1;7). (**A**) G-banding using trypsin and Giemsa staining (GTG banding) with marked breakpoints (UV–Vis microscope, Olympus BX41, magnification 1000×, software: Ikaros MetaSystems); (**B**) fluorescent in situ hybridization (FISH) staining, including various probe combinations (specific for: centromeres (cen), subtelomeres (ter), and whole chromosomes (wcp)), and multicolor FISH (mFISH)) (fluorescent microscope Zeiss AxioImager D1, magnification 1000×; software: ISIS MetaSystems); (**C**) a schematic representation of the bacterial artificial chromosome (BAC)-FISH) samples used (green—normal location; red—translocated), with marked breakpoint positions (arrows).

The characteristics of chromosomes involved in CCR are shown in Figure 1, including: partial karyotypes from G-banding using trypsin and Giemsa staining (GTG banding), results from fluorescent in situ hybridization (FISH) and bacterial artificial chromosome-FISH (BAC-FISH), and ideograms of the chromosomes with marked breakpoint localizations. Multicolor FISH (mFISH) analysis excluded other chromosomal rearrangements. Detailed cytogenetic analysis showed that chromosome 1 had two breakpoints: (i) at the short arm 1p, the suggested breakpoint region was 1p21 (the GTG resolution showed band 1p21 as a dark band, while BAC-FISH revealed that the breakpoint was down to 1p21.3 locus 94,630,298), and (ii) at the long arm 1q in a band 1q42.3 (locus between 233,745,426—the normal position; and 234,256,411—translocated on 1p). In the case of chromosome 7, only one breakpoint was found in long arm 7p in a band 7p14.3 (between 31,667,326—translocated on 1q; and 34,388,646—the normal position). A fragment of the arm of 7p was translocated into 1q42.3, while the terminal fragment of 1q was translocated into 1p21. Thus, the chromosome description was established as: der(1)(1qter->1q42.3::1p21->1q42.3::7p14.3->7pter), der(7)(1pter->1p2 1::7p14.3->7qter), for both brothers and for their mother.

**Figure 2:**
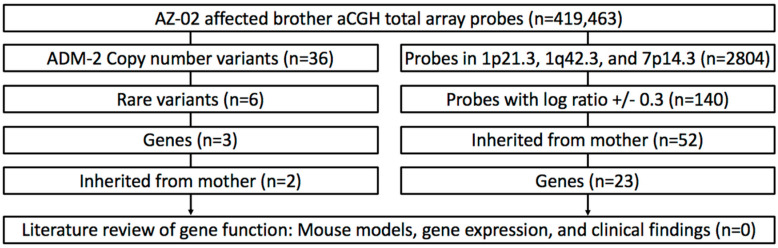
Workflow for genomic copy number variation (CNV) analysis of array comparative genomic hybridization (aCGH) data in the evaluated family. The major steps are shown in the left panel: (1) Low quality and noise probes removed, keeping real CNVs as described in the ADM-2 protocol in Materials and Methods (*n* = 36). (2) The confirmed variants were evaluated for rarity by a review of the database of genomic variants (DGV) for the reported CNV findings in healthy individuals (*n* = 6). CNVs that were reported more than five times in the DGV (fully overlapped and were the same type, amplification or deletion) were considered “polymorphic” variants and were excluded. (3) CNVs inherited from the mother were given higher priority (*n* = 2). (4) Rare CNVs with known genes were considered in a biological role (*n* = 1). (5) A gene review for gene function and testis-specific tissue expression was performed using the PubMed, OMIM, MGI, BioGPS, Unigene, and Aceview databases (*n* = 0). In the right panel, the major steps of the analysis of probes in the translocation regions are shown: (1) The probes were reviewed using the ADM-2 protocol for one probe (*n* = 2804). (2) All single probes with a log ratio of > 0.3 and < −0.3 were analyzed (*n* = 140). (3) CNVs that were also found in the mother were prioritized (*n* = 52). (4) CNVs for the genes analyzed (*n* = 23) and (5) rare genes were reviewed by a detailed literature and database search, as described above.

To identify potential unbalanced regions of the translocation and contributing genomic aberrations elsewhere, array comparative genomic hybridization (aCGH) experiments in the azoospermia patient (brother-1) and his mother were performed. Using a standard genome-wide 400 K array, high quality comparative genomic hybridization (CGH) data were obtained. Following standard genomic copy number variation (CNV) analysis of the aCGH data, 36 unique genomic CNVs were identified (Figure 2, Table 1). The steps included removing polymorphic variants via filtering the CNVs identified in the database of genomic variants (DGV db) (n = 5), annotation analysis for genes found in the remaining CNVs (n = 3) (Supplementary Table S2), and, finally, removal of those that did not show testis-specific expression and/or a plausible biological role in reproduction.

The final three nonpolymorphic CNVs in known genes: *ASMT* (acetylserotonin methyltransferase; OMIM: 300015; RefSeq: NM_004043.2), *GARNL3* (GTPase activating Rap/RanGAP domain like 3; RefSeq: NM_032293.5), and *SESTD1* (SEC14 and spectrin domain containing 1; RefSeq: NM_178123.5), were ruled out due to unlikely biological functions and a nonspecific testis expression profile. Thus, all identified candidate CNVs were ruled out (Supplementary Table S3). In addition, three breakpoint regions of the translocation were analyzed (Supplementary Figure S1) using a higher resolution analysis with individual CGH probes. For all breakpoint regions, 2804 probes were identified (Supplementary Tables S4 and S5). Respectively, each region’s probe resolution was calculated, ranging between 6 and 8 kb.

Next, we performed a revision of the single probes with amplification/deletion signal levels that had the potential to indicate variants too small to be detected by the aberration detection method 2 (ADM-2) algorithm. We identified 23 nonpolymorphic potential small CNVs with known genes. However, CNV filtering following the same biological annotation algorithm for confirmed CNVs did not reveal CNVs with biologically plausible genes (Supplementary Table S3). In addition, independent analysis of the CGH probe resolution (<10 kb) and RefSeq genes located in the translocation breakpoint regions ruled out the possibility of missing significant CNVs at the gene level resolution.

**Figure 3:**
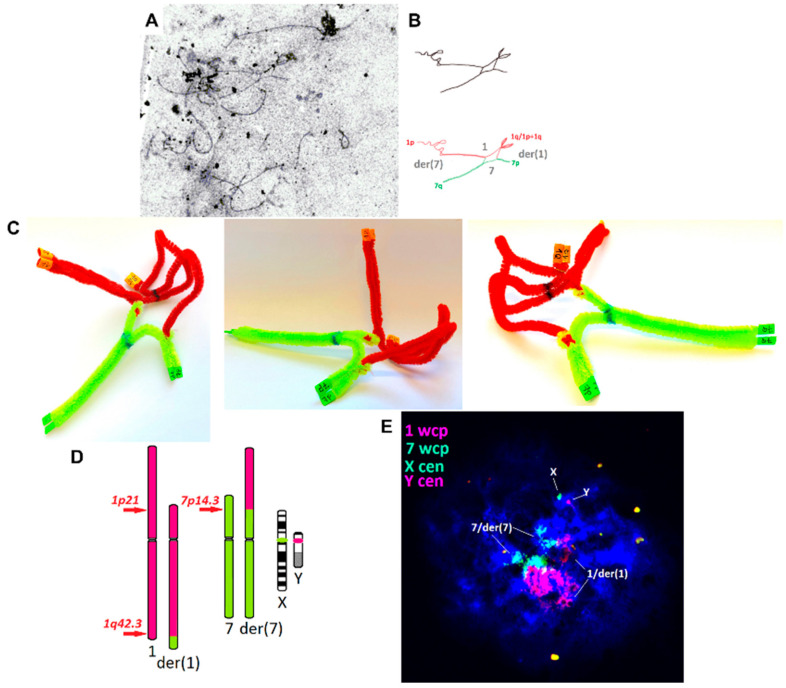
Analysis of the chromosomes at the spermatocyte II stage (brother-1 with azoospermia). (**A**) Synaptonemal complex staining (silver staining; UV–Vis microscope Olympus BX41, magnification 1000×, software: CellB Olympus); (**B**) a schematic suggestion of the involvement of chromosomes 1 and 7 in a meiotic quadrivalent form, following the length of the observed fragments; (**C**) a 3D model of CCR chromosome pairing, following the length of the normal and derivative chromosomes; red—chromosome 1; green—chromosome 7; navy blue—centromeres; brown—near the 1q–1p21 breakpoint; (**D**) the FISH staining scheme for the analysis of the association between XY-bivalent and tetravalent 1/7/der(1)/der(7); (**E**) the FISH result on spermatocyte II showing no association between XY-bivalent and tetravalent 1/7/der(1)/der(7) (fluorescent microscope Zeiss AxioImager D1, magnification 1000×; software: ISIS MetaSystems).

The synaptonemal complex analysis in brother-1 presented a spatial chromosomal formation curled into a ball (Figure 3A–C), which allowed the conjugation of proper fragments during meiosis. In only 1 of 53 (2%) analyzed cells, were chromosomes forming quadrivalents visible (Figure 3A), with a conjugation between the ends of the chromosomes involved (Figure 3B,C). Both the picture and a model also showed incomplete conjugation of chromosomal fragments. The quadrivalent figure was drawn following the proportions of the proper chromosome fragments (Figure 3B). The geometry was also confirmed by the 3D model of the synaptonemal complex (Figure 3C). No association between the CCR chromosomes and sex bivalence was observed (an example of FISH staining presented in Figure 3E).

**Figure 4:**
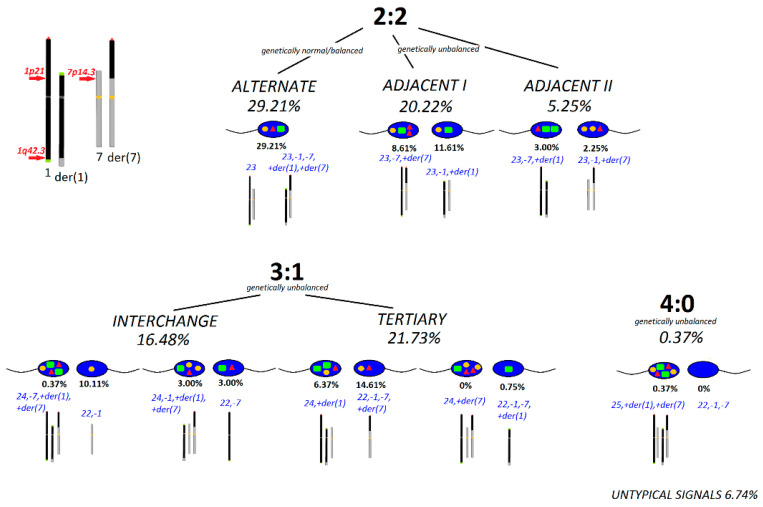
A schematic FISH representation of the labelling and frequency of particular segregation types for meiotic segregation pattern analysis performed for the spermatozoa of brother-2. FISH probes used: subtelomere specific for 1pter (red triangle), for 1qter (green square), and a centromere specific for chromosome 7 (yellow ellipse = red + green) (as described in the Materials and Methods section). The number of evaluated spermatozoa: *n* = 267.

In brother-2, the evaluation of the meiotic segregation pattern was performed on ejaculated spermatozoa. A schematic FISH representation of the labelling and the frequencies of particular segregation types is shown in Figure 4. The frequency of genetically normal/balanced spermatozoa was 29.21%. Among genetically unbalanced gametes, the highest percentage was observed after segregation, 3:1 type (38.21%), followed by adjacent I (20.22%), adjacent II (5.25%), and 4:0 (0.37%). Sperm cells with untypical FISH signals had a frequency of 6.74%.

## 3. Discussion

In this study, we investigated the molecular characteristics of a genetically balanced exceptional CCR involving chromosome 1 (with two breakpoints) and 7 (one breakpoint) found in the family of two infertile brothers and their mother. We narrowed down the breakpoint positions using FISH experiments with commercially available FISH probes, and experimental BAC probes for selected regions of interest. Additionally, we performed aCGH evaluation to determine whether, in the regions of the breakpoints, any CNVs with known genes crucial for spermatogenesis were present, but no plausible candidates were found.

Within more than 250 of papers related to the CCR cases published so far, 161 cases concerned male CCR carriers. To our knowledge, only 64 cases were evaluated for reproductive problems (including one Klinefelter case) (Supplementary Table S1). In 31 cases, the spermatozoa were able to fertilize, leading to reproductive failures and reproductive successes, while the other 31 patients remained infertile. In two CCR cases, the fertility history was unknown. Two infertile CCR cases performed the intracytoplasmic sperm injection (ICSI) procedure, which resulted in genetically balanced offspring with CCRs. Within the group of patients with reproductive failures, in 25 cases, repeated abortions (RAs), miscarriages, and/or multiple congenital abnormalities (MCAs) were observed in the offspring. Only four CCR carriers had no reproductive failures and fathered children with normal (2), abnormal (1), or unknown (1) karyotypes (Supplementary Table S1). When considering the semen parameters among those 64 male CCR carriers, in 15 cases (23.4%) azoospermia was observed, followed by cryptozoospermia and oligozoospermia (C, O; 3/64—4.8%, each), oligoasthenozoospermia (OA; 4/64—6.3%), oligoasthenoteratozoospermia (OAT; 7/64—10.9%), isolated asthenozoospermia (A; 1/64—1.6%), or others. Normal semen parameters were noted for nine males only (N; 14.1%), while in 10 cases, the seminal analysis was not performed (Supplementary Table S1).

As mentioned previously, there are four types of CCR depending on the number of chromosomes and breakpoints involved in the rearrangement. Within the 63 reviewed cases (except the Klinefelter case), type I—the simplest and most common, was found in 39.7% of CCR males with reproductive problems (Supplementary Table S1). Type II was observed in 30.2% of cases (Supplementary Table S1). Our CCR case described in the present study belongs to the type II group, with two breakpoints on chromosome 1 and one breakpoint on chromosome 7. Type III—the coincidental presence of two or three chromosomal translocations, was noted in 17.7% of CCR males with reproductive problems (Supplementary Table S1). The last type of CCR, type IV—the genetically imbalanced type, was observed in 9.5% of CCR males with reproductive failures.

Another aspect of CCRs is the involvement frequency of particular chromosomes in CCR. When considering the data concerning 63 CCR males with reproductive failures (Supplementary Table S1), we found that the total number of chromosomal breaks was 208. Among these, 19 breakpoints were found in chromosome 1, followed by chromosome 9 (17), chromosomes 13 and 3 (17), and chromosomes and 14 and 4 (16). No breakpoints were found for chromosomes: X, 17, and 20. Such observations may lead to two conclusions. First, the larger the chromosome that is involved in the CCR, the higher the probability for rearrangement. Additionally, there is a correlation between the group of chromosomes involved and the proper type of meiotic segregation [14]. Chromosomes from groups A–C (the large ones) were segregated mostly in adjacent I mode (76%), followed by the 4:2 mode with chromosomes from the D–G groups (the small ones).

In CCR carriers with recurrent abortions, the involvement of chromosomes from groups A–C was often observed (84%). Second, chromosome abnormalities have been widely observed in cases with reproductive problems, which are prone to breakpoint appearances. The best example is chromosome 9 and the acrocentric ones for which the involvement in CCR is clearly linked to the 4:2 segregation mode [14]. Our CCR case described in the present study concerned chromosomes 1 and 7. As described above, chromosome 1 is the most prone to breakpoint occurrence, while chromosome 7 is in the group of medium risk with 11 breakpoints noted (Supplementary Table S1). Thus, the involvement of both chromosomes in CCR is not surprising. The mean number of breaks per rearrangement did not differ between fertile and infertile male CCR carriers, even considering that the risk of reproductive failures and infertility increased with the complexity of the CCR (Supplementary Table S1, [14,30]).

The origin of the CCRs in the majority of the reviewed cases (36/64) was unknown, followed by 16 *de novo* cases, seven maternally inherited (also our cases described in this study), two paternally inherited, two mixed, and one inherited but not defined. Certain familial CCRs can change from one generation to another. For example, carriers of double two-way translocations may lead to a more complex rearrangement in their offspring [31,32,33,34], or carriers of, e.g., three-way translocations may father children with two simple reciprocal translocations [35]. Such rebuilding and rearrangement, leading to simpler or more complicated aberrations, arises in approximately 45% of CCR cases [17]. An example is a case of a fertile CCR carrier of der(Y;15),rob(13;14) who inherited his CCR from both parents: der(Y) form his father and rob(13;14) from his mother, and fathered by himself a healthy daughter (46,XX) [36]. In the rest of the CCR cases, the rearrangement remained the same (also as described in our study).

Another interesting point is the varied fertility status in family members with the same chromosomal aberration (i.e., fathers and sons, brothers, or cousins). Johanisson et al. described a familial case of family members with the same CCR t(9;12;13), including the father, three daughters, and two sons [37]. Among the sons, one was infertile, while the second one was subfertile. Both brothers were evaluated meiotically according to the possible associations between hexavalent and XY bivalent. The results for both brothers were similar—in 80–90% of spermatocytes, no association was found. The reasons for the different phenotypes remained unknown [37]. Another example is a case of two brothers and a cousin, where brother 1 was a CCR carrier of double two-way translocation t(8;9), t(1;16) and revealed infertility, while his brother and cousin carried the same simple RCT t(8;9) and had progeny [34]. Likely, the accumulation of two rearrangements simultaneously, followed by the increased frequency of genetically unbalanced spermatozoa, resulted in a lack of conception. Similarly, in RCT carriers, there are also known cases of varied fertility statuses in male family members [38,39,40,41,42,43,44,45,46]. The reasons for such situation remain unclear. The answer may be hidden in the genomes of particular patients, and next-generation sequencing (NGS) methods will allow researchers to identify them.

Next, we discuss the inheriting of CCR between sexes, i.e., from a mother to son, and the differences in the (in)fertility between parents and their offspring. An example is a case of an infertile oligoasthenozoospermic male with CCR involving chromosomes 1, 3, and 13, with a lack of conception [47]. He inherited CCR from his mother who had no fertility problems. Similarly, in a case of t(1;3;6) described by Hornak et al. [48], the normozoospermic CCR carrier was infertile, while his mother and her sister (also CCR carriers) were fertile [48]. The detailed genomic explanation in this case remained unknown; however, it was able to be linked to the familial transmission from mother to son.

Oogenesis lacks chromosome-mediated checkpoint control at the metaphase/anaphase stage, due to the absence of a sex vesicle (a unique element for spermatogenesis, representing a specific chromatin state of the sex chromosomes) [49]. This leads to the high rate of meiotic nondisjunction in females, and is responsible for the increased chromosomal error rate in oogenesis [49,50,51]. The carriership of CCR mostly leads to chromosomal asynapsed regions, which in males are prone to the association of XY bivalent and then to the disruption of spermatogenesis [26,52]. Heterozygous females are predominantly fertile and have phenotypically normal offspring, while male carriers are subfertile or even sterile. Our case presented in this study seemed to confirm this observation. Namely, the mother of the brothers was fertile, even with two reproductive failures in her reproductive history, but the both evaluated brothers remained infertile due to the lack or extremely low number of spermatozoa.

In the case of the brother B1 with azoospermia, as described in the present study, synaptonemal complex analysis revealed a spatial curled configuration of the quadrivalent with the asynapsed interstitial fragments. However, no association with XY bivalent was observed. Of course, such associations cannot be excluded due to the small number of cells analyzed (*n* = 24). The curled formation of the quadrivalent was so sophisticated that it hindered the procession of the further steps of meiosis; thus, we can assume that the spermatogenetic block appeared at the pachytene stage of meiosis.

What about the cryptozoospermia phenotype of the brother B2 with the same CCR? In this case, the spermatogenesis went further in a very restricted manner—few sperm cells were found in the ejaculate, and they were genetically imbalanced (71%). Factors such as the age or environmental items were excluded as potentially influencing the state of spermatogenesis in both brothers. Both of them worked in normal conditions and were similar in age. Additionally, both brothers performed several sperm analyses within the last 7 years, but the results were the same: azoospermia (B1) and cryptozoospermia (B2). We can suppose two reasons for that.

First, various levels of association between the asynapsed regions and XY body between the two brothers. However, (i) in brother B1 no associations were observed, but the number of cells analyzed was too small to conclude without hesitation. Additionally, we had no possibility to analyze material from the testis from the brother B2. Therefore, we can neither accept nor exclude this hypothesis due to the lack of material. The limited volume of testicular oligobiopsy derived from brother-1 and the lack of such sample from brother-2 constituted the primary limiting factor for performing any additional examination of meiosis. Testicular oligobiopsy is difficult to accept in many individuals. Additionally, in circumstances in which there are some spermatozoa available in the ejaculate, potentially allowing for *in vitro* fertilization (IVF) participation (as in brother-2 with cryptozoospermia), there is no recommendation for gonadal open biopsy sampling.

The second reason is that the answer is likely hidden in genetic mutations, independent from the cytogenetic characteristics of the described cases. To come closer to the answer, we performed aCGH screening, which revealed variations in 26 genes, including three rare variants in genes: *ASMT, GARNL3*, and *SESTD1.* However, further filtering according to established criteria, excluded the potential gene candidates for being responsible for various phenotypes between both brothers. Thus, the problem concerning the differences in phenotypes remained unsolved. aCGH analysis could not detect smaller intergenic CNV of few kb or smaller, such as indels of single exons or a few nucleotides. This suggests the need for whole exome or genome sequencing (WES/WGS) to investigate the presented familial case further, especially as genomic sequencing studies suggested that 85% of known genetic variants causing diseases are represented in the human coding sequence by point mutations causing the respective disorders [53,54,55].

In summary, chromosomal aberrations mostly do not affect the phenotype of the carrier, in contrast to the disruptive influence on spermatogenesis, which affects the gamete quantity (oligo-, azoospermia) and/or quality (generation of chromosomally abnormal sperm) of the carrier. Therefore, the detailed characteristics of each CCR case constitute a highly valuable source of data for genetic counselling in cases with reproductive failures. This also supports the necessity of careful genomic analysis in cases with the possibility of novel point mutations and supports the addition of the new information to existing databases of male infertility.

## 4. Materials and Methods

### 4.1. Patients

**Figure 5:**
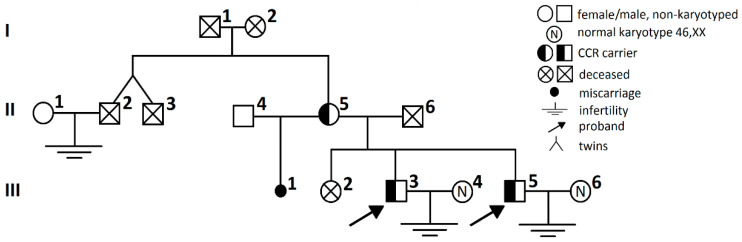
Pedigree information for the CCR t(1;7) family. Two probands are indicated for the brothers (marked with arrows): with azoospermia (brother-1, III-3) and 10 years of lasting infertility, and with cryptozoospermia (brother-2, III-5) and 3 years of lasting infertility. Their mother (II-5), a carrier of the same CCR, revealed one miscarriage at the 12th week of gestation (III-1), and one child death at nine months (III-2), likely due to myelodysplasia (but this was not documented). An infertility case was also noted in another family member (II-2, 20 years of a lack of conception).

Pedigree information for the family CCR case is presented in Figure 5. The samples analyzed consisted of material from three members of one family: the mother (M, II-5, age 59), brother-1 (III-3, age 31) with azoospermia, and brother-2 with cryptozoospermia (III-5, age 27). All of them were carriers of a non-mosaic complex chromosome rearrangement involving two chromosomes and three breakpoints (chromosome 1—two breakpoints, chromosome 7—one breakpoint). The chromosomes involved in CCR and the breakpoints were identified by classic cytogenetic methods, including GTG banding, and then confirmed with FISH. Both brothers presented infertility (lack of conception; their wives have a normal karyotype 46,XX), and their multiple semen analyses demonstrated nonobstructive azoospermia (brother-1; no spermatozoa, semen volume: 1.5–2.0 mL) and cryptozoospermia (brother-2; sperm concentration: 0.1 × 10^6^/mL, semen volume: 1.8–2.0 mL, single round cells after centrifugation) (according to guidelines of the World Health Organization WHO, 2010, [56]).

No Y chromosome microdeletions in the sex-determining region Y (*SRY*, RefSeq: NM_003140) and azoospermia factor region (AZF) (according to European Academy of Andrology (EAA) guidelines), and no mutations in the *CFTR* gene (RefSeq: NM_000492) were identified. Hormone evaluation was performed only in brother-1 and revealed increased levels of follicle-stimulating hormone (FSH; 15.0; reference value 1–14.0 mo/L) and luteinizing hormone (LH; 12.0; 0.7–7.4 mo/L), while the level of testosterone was normal (4.8; 0.3–12.0 ng/mL). The biological samples included DNA extracted from the blood (the mother and both brothers), lymphocytes fixed according to classical 3:1 ice-cold methanol:acetic acid fixative (the mother and both brothers), testicular oligobiopsy, fixed with 4% paraformaldehyde solution (PFA/1× PBS) for synaptonemal complex analysis (brother-1), and ejaculate, fixed with 3:1 ice-cold methanol:acetic acid fixative (brother-2). All analyzed patients were notified of the purpose of the research and written informed consent was obtained, according to guidelines of the Local Bioethical Committee, Poznan University of Medical Sciences (approval no. 772/15; approved on 01 October 2015).

### 4.2. Fluorescence In Situ Hybridization (FISH)

The FISH experiments were prepared with combinations of directly labeled probes and/or with BAC probes prepared for the proper chromosomal regions. FISH probe combinations were used for:

(i) The characterization of chromosomes involved in CCR. Centromere-specific: 1cen (*locus* D1Z1, catalogue number LPE01R/G) and 7 cen (D7Z1, LPE07R/G). Subtelomere: 1pter (clone CEB108, LPT01pR), 1qter (clone 160H23, LPT01qG), and 7pter (clone 109a6, LPT07pR, Cytocell, UK). Whole chromosome painting: 1wcp (catalogue number XCP1R), 7wcp (XCP7G), and mFISH (multicolour FISH) (MetaSystems, Altlussheim, Germany). For each combination, at least 50 metaphase plates were analyzed, and 1000 interphase cells (FISH with cen/subtel probes) were counted to exclude the mosaicism.
(ii) The translocation breakpoint analysis—BAC probes for the proper chromosomal regions are listed in Supplementary Table S6 and in Figure 1. BAC clones were chosen from the RPCI-11 library collection (BACPAC Resource Center, the Children’s Hospital Oakland Research Institute) based on hg19 (human genome reference GRCh37 h37). At least 10 metaphase plates were analyzed for each round of FISH.
(iii) The analysis of the association between the chromosomes involved in CCR and sex chromosome bivalent (spermatocytes II from the testicular oligobiopsy of brother-1). wcp and cen-specific probes: 1wcp (XCP1R), 7wcp (XCP7G), Xcen (DXZ1, LPE0XG), and Ycen (DYZ3, LPE0YcR) (MetaSystems, Germany; Cytocell, UK); *n* = 24.
(iv) The meiotic segregation pattern in the sperm cells from the ejaculate of brother-2. A 3-colour combination of cen-specific and subtel-specific probes: 1pter (LPT01pR), 1qter (LPT01qG), and 7cen (D7Z1, LPE07R/G) (Cytocell, UK); *n* = 267.

### 4.3. BAC Preparation

The BAC DNA was isolated using Qiaprep Spin Miniprep kit and protocol (Qiagen). BAC labelling was prepared according to the manufacturer’s instructions with a labelling kit (Platinum Bright: Nucleic Acid Labeling Kit, Kreatech; catalogue number: GLK-001 for green, GLK-002 for red/orange). Briefly, 1 μg of BAC-DNA was mixed with 2 μL of ULS (universal linkage system labelling DNA by binding to the N7 position of the guanine), and 2 μL of 10× labelling solution, to a final volume of 20 μL. Next, the prepared mix was incubated at 85 °C for 30 min, and then put on ice and spun down (6000 rpm, 1 min). Labelled BAC was purified from the excess dye using a spin column. Then, repetitive α-satellite sequences were blocked, and non-specific binding was reduced by adding 25× excess of C_0_T DNA (Kreatech) and ssDNA (Invitrogen; catalogue number 15632-011). The BAC sample was precipitated with 1/4 vol. of 10 M NH_4_Ac and 2.5 vol. of 100% EtOH, −20 °C, overnight. Next, the sample was centrifuged (15000 rpm, 30 min., 0 °C), and the resulting pellet was air-dried. The BAC-DNA was dissolved in 50% hybridization solution and frozen at −20 °C until the FISH procedure.

### 4.4. Hybridization

The fixed semen sample from brother-2 was spread onto slides, washed in PBS, and incubated in a decondensation solution (10 mM DTT, 100 mM TRIS-HCl; pH 8.5, 43 °C) for 7 min. Next, the slides were rinsed in 2× SSC (pH 7.0), air-dried, and then stored in a freezer at −20 °C until the FISH procedure. Fixed lymphocyte cultures were spread onto slides directly before FISH. FISH was performed following the manufacturer’s protocol (Cytocell, Cambridge, UK) with modifications described previously [57]. The hybridization mixtures contained various volumes of probes depending on their specificity: centromere—2.0 µL; subtelomere—3.0 µL; wcp/mFISH—10.0 µL; and BAC—5.0 µL. If needed, the mixes were filled with hybridization solution to a final volume of 10 or 20 µL. The FISH efficiency was approximately 98%. For analysis, a Zeiss AxioImager D1 microscope equipped with the necessary filters (DAPI/FITC/SpO/TR/Cy5/DEAC/Triple) and objectives (20×, 100× immersion) was used. Images were acquired with a CCD camera (Jenoptik, Germany) and processed using ISIS software (MetaSystems, Altlussheim, Germany).

### 4.5. Genomic Microarray CGH

To test for potential genomic aberrations, genome-wide aCGH was performed using SurePrint G3 Human CGH 2 × 400 k Oligo Microarrays (Agilent Technologies, Santa Clara, CA, USA). CGH analysis was performed according to the manufacturer’s protocol (Agilent Technologies). The array design featured 420,288 total distinct biological features, or probes. Briefly, genomic DNA for brother-1 and the mother was extracted from the patients’ peripheral blood leukocytes (Puregene; Qiagen). Reference male DNA was purchased from Promega (Madison, WI). Experimental DNA was enzymatically fragmented and labeled with fluorescent dye Cyanine-5; the reference DNA was labeled with Cyanine-3 dye. The labeled DNA was hybridized to the CGH probes for 40 h at 66 °C. After washing, the array slides were scanned (SureScan Microarray C scanner, Agilent) and analyzed using CytoGenomic Workbench software (Agilent). The CGH array quality was assessed as good, since 419,463 biological features (99.8%) were identified successfully.

Copy number variant (CNV) calling was performed using the ADM-2 protocol with a minimal region of three consecutive probes, a mean log ratio >0.25 or <−0.25, and a direct signal intensity range of 100–1000 units. Variant calls that met the protocol criteria were reviewed initially to exclude variants of uncertain quality, such as calls with large areas of missing probe coverage (>100 kb). To verify, if the resultant CNVs were polymorphic, the CNVs were checked for frequency in the DGV database (Database of Genomic Variants, The Centre for Applied Genomics). Variants that were fully overlapped with >5 reported CNVs of the same type (amplification or deletion) in the DGV were considered “polymorphic.” Gene function annotation was performed with the OMIM (Online Mendelian Inheritance in Man), MGI (Mouse Genome Informatics, Jackson Laboratories), and RefSeq databases. The expression annotation was done with BioGPS (Scripps Research Institute) and Aceview (NCBI).

### 4.6. Synaptonemal Complex Analysis

The evaluation of synaptonemal complexes was performed on secondary spermatocytes from a testicular oligobiopsy of B1. After the collection of the testis sample via oligobiopsy, germline cells were placed in a Petri dish with 10 drops of 1× PBS (pH 7.4; Lonza, Walkersville, MD, USA). Next, three drops of 0.4% KCl were added and the sample was macerated with forceps to obtain a suspension of single cells (all under a light microscope inspection). The resuspended cells were placed onto slides, air-dried, and fixed with 4% PFA/1× PBS for 10 min. at room temperature. Next, the slides were rinsed with 0.5% Triton X-100 (Sigma), air-dried, and incubated in 70% EtOH for 10 min. The fixed biopsy samples were stored at 4 °C until further use.

The synaptonemal complexes were visualized using the silver staining technique described previously [58,59]. Briefly, the slide was heated at 80 °C for 5 min. Then, freshly made buffers were applied: four drops of 50% AgNO_3_/water and two drops of a solution of 1% formic acid in 2% gelatin/water. The covered sample was incubated at 80 °C for 2–3 min., until the silver precipitated (dark brown color). Next, the slide was rinsed in distilled water and incubated in 4% Giemsy staining solution (Merck) for 10–12 min. After rinsing in water and air-drying, our microscopic analysis was performed (Olympus BX41, magnification 1000×, oil-immersion, software: CellSense Dimensions, Olympus). The number of analyzed cells was *n* = 53.

## Figures and Tables

**Table 1:** The CNV analysis report summary of the aCGH results for the AZ patient. Variants were identified and annotated using Cytogenomics software with the ADM-2 protocol criteria. All variants were then manually reviewed and categorized using the process described in Figure 3. The frequency was either polymorphic, labeled “pol,” or rare, based on the DGV database (The Centre for Applied Genomics). All variants were compared to the mother’s CGH results and variants were identified as maternally inherited (“Yes”) or not (“No”).

Event	Chr	Cytoband	#Probes	Log Ratio	Annotations	Gene Effect	Inherited from Mother
1	chr1:152556449–152581944	q21.3	6	0.617541	*LCE3C*	pol	Yes
2	chr1:196742735–196796220	q31.3	5	−0.978775	*CFHR3*, *CFHR1*	pol	No
3	chr2:34697718–34730142	p22.3	7	−0.636087		pol	Yes
4	chr2:78709955–78721280	p12	4	−0.852013		pol	No
5	chr2:87392136–87801877	p11.2	11	0.508958	*NCRNA00152*	pol	Yes
6	chr2:132205346–132217492	q21.1	3	−0.809717		pol	No
7	chr2:180069605–180070733	q31.2	3	1.002822	***SESTD1*, 1MB dup**	rare	Yes
8	chr3:162556223–162619141	q26.1	7	0.471521		pol	No
9	chr4:69387056–69483277	q13.2	12	−0.742031	*UGT2B17*, *UGT2B15*	pol	No
10	chr5:17345455–17353452	p15.1	3	−0.926486		pol	Yes
11	chr5:140223256–140236399	q31.3	4	−0.878128	*PCDHA1*, *PCDHA2*, *PCDHA3*	pol	Yes
12	chr6:259881–287425	p25.3	6	0.523226		pol	Yes
13	chr6:32450699–32493043	p21.32	6	−1.853271	*HLA-DRB5*	pol	No
14	chr6:165725547–165737665	q27	3	0.948835		rare	No
15	chr7:141750430–141792094	q34	9	−0.511123	*MGAM*,	pol	Yes
16	chr8:39234992–39386158	p11.22	28	0.821278	*ADAM5P*, *ADAM3A*, 151 kb dup	pol	Yes
17	chr9:130041553–130145721	q33.3	23	−0.981719	***GARNL3*, 104 kb del, het**	rare	No
18	chr11:18949929–18960666	p15.1	3	0.804263	*MRGPRX1*, 10 kb dup het	pol	No
19	chr11:25635357–25764082	p14.3	9	0.689271		rare	Yes
20	chr11:55368154–55450788	q11	16	0.436620	*OR4C11*, *OR4P4*, *OR4S2*	pol	Yes
21	chr12:9637323–9718846	p13.31	11	1.538928		pol	Yes
22	chr12:10583558–10593748	p13.2	3	−0.864549	*KLRC2*,10 kb del het, gene cluster	pol	No
23	chr12:11218244–11225675	p13.2	3	−1.376538	*PRR4*, *PRH1*	pol	No
24	chr12:11230835–11249210	p13.2	3	−4.773687	*PRR4*, *PRH1*, *TAS2R43*	pol	Yes
26	chr14:74001651–74012568	q24.3	3	−5.04754	*HEATR4*, *ACOT1*, 1.1MB del	pol	Yes
27	chr16:70174866–70193942	q22.1	4	−0.996207	*PDPR*	pol	No
28	chr16:74394080–74407341	q23.1	3	−0.943902	*LOC283922*	pol	No
29	chr16:78372097–78381281	q23.1	3	−5.242076	*WWOX*	pol	No
30	chr17:34437475–34475514	q12	7	0.847688		pol	Yes
31	chr19:53522243–53550020	q13.41	6	0.783781		pol	No
32	chr20:1563715–1577359	p13	4	−3.864894	*SIRPB1*, 13 kb homo del	pol	Yes
33	chr22:18889039–19010562	q11.21	20	0.522787	*DGCR6*, *PRODH*, *DGCR5*…	pol	Yes
34	chr22:24347959–24395353	q11.23	10	0.607834	*LOC391322*, *GSTT1*, *GSTTP2*	pol	No
35	chr22:25664618–25919542	q11.23–q12.1	54	0.578645	*IGLL3*, *LRP5L*	pol	No
36	chrX:1731610–1752284	p22.33	10	0.513510	***ASMT***	rare	No

## Supplementary Materials

Supplementary Materials can be found at https://www.mdpi.com/1422-0067/21/12/4559/s1. Supplementary Figure S1. CGH probe position on chromosomes 1 and 7. Supplementary Table S1 Male CCR carriers with evaluated reproductive status, according to the literature data published so far. Supplementary Table S2 Non-polymorphic variants found in aCGH screening of brother-1 and mother samples. Supplementary Table S3. Final result of aCGH screening—list of genes found, incl. their role. Supplementary Table S4 Coordinates of 3 breakpoint regions of the translocation analyzed using a higher resolution analysis with individual aCGH probes. Supplementary Table S5 aCGH results obtained for 3 breakpoint regions of the translocation. Supplementary Table S6 BAC clones used in breakpoint mapping of the chromosomes involved in CCR t(1;7).
