# Supplementary material for: Familial Infertility (Azoospermia and Cryptozoospermia) in Two Brothers—Carriers of t(1;7) Complex Chromosomal Rearrangement (CCR): Molecular Cytogenetic Analysis
Source: Int J Mol Sci. 2020 Jun 26;21(12):4559. doi: 10.3390/ijms21124559 (PMC7349667; doi:10.3390/ijms21124559)
Supplement: Supplementary file 1 [file ijms-21-04559-s001.zip › Supplementary Table 1.docx]

**Supplementary Table S1** Male CCR carriers with evaluated reproductive status, according to the literature data published so far.

azoo – azoospermia, crypto – cryptozoospermia, necro – necrozoospermia, O – oligozoospermia, OA – oligoasthenozoospermia, OAT – oligoasthenoteratozoospermia, OATLN – oligoasthenoteratoleucocytonecrozoospermia, A – asthenozoospermia, AT – asthenoteratozoospermia, N – normozoospermia, NA – non available (not evaluated), RA – recurrent abortion, MCA – multiple congenital abnormalities, ICSI – intracytoplasmic sperm injection, RCT – reciprocal chromosome translocation, mat – inherited maternally, pat – inherited paternally, FISH – fluorescent *in situ* hybridization

| **No.** | **Karyotype** | **inheritance** | **type of CCR** | **methods** | **Seminology** | **reproductive history** | **reference** |
| --- | --- | --- | --- | --- | --- | --- | --- |
| 1 | 47,XXY,t(1;3;5)(p22;q29;q22) [Klinefelter syndrome] | mat | - | classic banding | azoo | infertility | [1] |
| 2 | 46,XY,der(1)(1pter->1q31.3::9q13->9qter),der(3)  (9pter->9p21.3::3p14->3q12::14q13->14qter),der(9)  (3pter->3p24::3q24->3q12::3p24->3p14::9p21.3->  9q13::1q31.1->1qter),der(14)(14pter->14q13::3q24->3qter) | - | II | classic banding, FISH | azoo | infertility | [2] |
| 3 | 46,XY,der(1)(9qter?9q22::1p32?1qter),der(4)(1pter?  1p32::13q32?13q14::4p14?4qter),der(9)(9pter?9q22::4p14?  4pter),der(13)(13pter?13q14::13q32?13qter) | - | II | classic banding, FISH | azoo | infertility | [3] |
| 4 | 46,XY,der(1)(5qter->5q14.3::15q21->15q26::1p13->1qter),  der(5)(5pter->5q14.3::1p13->1pter),  der(15)(pter->q21::q26->qter) | - | II | classic banding, FISH | azoo | infertility | [4] |
| 5 | 46,XY,t(1;10)(q43q44;q21q26.1),  ins(14;4)(q31.3;q23q33) | - | II | classic banding, FISH | azoo | infertility | [5] |
| 6 | 46,XY,t(2;19;22)(q11.2;p13.2;p11.2) | - | I | classic banding, FISH | azoo | infertility | [3] |
| 7 | 46,XY,der(3)(3pter->3p23::3q25.3->3p11.1::6q27->6qter),  der(6)(6pter->6q27::16q24->16qter),der(12)(12pter->  12q24.3::3q25.3->3qter),der(16)(16pter->16q24::3p11.1-> 3p23::12q24.3->12qter) | - | II | classic banding, FISH | azoo | infertility | [3] |
| 8 | 46,XY,t(3;16;8)(p26;q13;q21.2) | *de novo* | I | classic banding | azoo | infertility | [6] |
| 9 | 46,XY,t(5;1;10;12)(5qter->5p13::12q24->12qter;1pter-> 1q42::5p13->5pter;10pter->10q24::1q42->1qter;  12pter->12q24::10q24->10qter) | *de novo* | I | classic banding | azoo | infertility | [7] |
| 10 | 46,XY,t(5;7;9;13)(q11;p11;p15;q12;p12) | - | I | classic banding, FISH | azoo | infertility | [8] |
| 11 | 46,XY,t(9;13;14)(p22;q21.2;p13) | - | I | classic banding | azoo | infertility | [9] |
| 12 | 46,XY,t(9;13;21)(p22;q22;p11) | - | I | classic banding | azoo | infertility | [10] |
| 13 | 46,XY,der(11)(11pter->11q22),der(12)(12pter->12q13:  :11q22->11qter),der(21)(21qter->21p11:12q13->12qter) | - | I | classic banding | azoo | infertility | [11] |
| 14 | 46,XY,inv(12)(p13.31q23.2),ins(7;12)(p21.3;q23.2q12) | - | II | classic banding, FISH | azoo | Infertility; 5 sisters and4 brothers - nonkaryotyped | [12] |
| 15 | 46,Xder(Y)(Ypter->Yq11.23::12q21.2->12qter),der(12)  (12pter->12p11.2::12q21.2->12p11.2::15q13->15qter),  der(15)(15pter->15q13::Yq11.23->Yqter) | *de novo* | I | classic banding, FISH | azoo | infertility | [13] |
| 16 | 46,XY.ish t(1;4)(q42;q32),ins(1;11)(q41;q23q24),  ins(4;11)(q23;q14q23) | *de novo* | II | classic banding, FISH | azoo/crypto | ICSI: balanced CCR daughter | [14] |
| 17 | 46,XY,der(3)(14pter::3q10?3qter),  der(14)(3pter?3p11.1::14q21?14p11::14q21?14qter) | - | II | classic banding, FISH | crypto/necro | infertility (Sertoli cells only; BUT immotile sperm after centrifugation | [3] |
| 18 | 46,XY,der(7)t(7;13)(p14;q21),der(9)t(9;13)(p11.2;p21),  der(13)t(7;13)(q14;q21)t(9;13)(9p11.2;p21) | *de novo* | II | classic banding, FISH | crypto | infertility | [15] |
| 19 | 46,XY,der(1)(1qter->1p13.3::9q22.1->9qter),  der(6)(6pter->6q15::?1p13.3->?1p31.2::14q31->14qter), der(9)(9pter->9q22.1::6q15->6qter),  der(14)(14pter->14q31::1p31.2->1pter) | - | II | classic banding, FISH | O | infertility | [16] |
| 20 | 46,XY,der(2)(2qter->2p25.1::13q13->13q22::18q12.3-> 18qter),der(13)(13pter->13q13::2p25->2pter),  der(18)(18pter->18q12.3::13q22->13qter) | *de novo* | II | classic banding, FISH | O | RA | [17] |
| 21 | 46,XY,t(5;13;14)(q23;q21;q31)(5pter->5q23::14q31-> 14qter;13pter->13q21::5q23->5qter;  14pter->14q31::13q21->13qter) | - | I | classic banding, FISH | O | RA | [18] |
| 22 | 46,XY,der(1)(3qter->3q12::1p32->1q42::3p13->3pter),  der(3)(1pter->1p32::3p13->3q12::13q14.1->13qter),  der(13)(13pter->13q14.1::1q42->1qter) | mat | II | classic banding, FISH | OA | infertility | [19] |
| 23 | 46,XY,t(1;19;13)(1qter->1p31::13q31->13qter;19pter->19q13.2::1p31->1pter;13pter->13q31::19q13.2->19qter) | mat | I | classic banding, FISH | OA | infertility; ICSI: balanced CCR male fetus | [20] |
| 24 | 46,XY,t(3;4)(p21;q21),t(12;14)(q14;q31) | - | III | classic banding | OA | infertility | [21] |
| 25 | 44,X,der(Y),t(Y;15)(q12;q10)pat,  rob(13;14)(q10;q10)mat | pat/mat | III | classic banding | OA | fertile - 1 daughter 46,XX | [22] |
| 26 | 46,XY,der(1)(1qter->1p35.1::10q26.13->10qter),  der(2)(2pter->2q21.3::1p35.1->1pter),  der(10)(10pter->10q11.23::10q24.33->10q26.13:  :10q24.33->10q11.23::2q21.3->2qter) | - | II | classic banding, FISH | OAT | infertility | [23] |
| 27 | 46,XY,t(1;22;4)(p22.3;q11.1;q31.1) | - | I | classic banding | OAT | infertility | [24] |
| 28 | 46,XY,t(2;7;4)(q31;q34;q33) | - | I | classic banding, FISH | OAT | infertility | [3] |
| 29 | 46,XY,t(6;10;11)(q25.1;q24.3;q23.1) | mat | I | classic banding, FISH | OAT | infertility | [25] |
| 30 | 46,XY,der(9)t(9;12)(q32;p13),  der(12)(14qter->14q32::12p13->12q13::9q32->9qter), der(14)t(12;14)(q13;q32.2)/46,XY | *de novo* | II | classic banding, FISH | OAT | 2 children, secondary infertility | [26] |
| 31 | 44,XY,der(13;14)(q10;q10),der(21;22)(q10;q10) | - | III | classic banding, FISH | OAT | infertility | [27] |
| 32 | 46,XY,der(13)(t(13;18)(q22;q21.2)  ins(13;14)(q22;q24q32.1),del(14)(q24q32.1),  der(16)t(16;13)(p12.3;q22),der(16;18)(p12.3;q21.2) | *de novo* | IV | classic banding, FISH | OAT | infertility; infertile twin brother - CCR carrier; 2 fertile brothers - non-karyotyped | [28] |
| 33 | 46,XY,der(2)(12pter->12p13.1::2p12.1->2qter), der(4)ins(4;2)(p15.1;p23.3-p13.3),  der(12)(2pter->2p24::12p12.3->12qter)/46,XY | *de novo* | II | classic banding, FISH | OATLN | 1 child | [26] |
| 34 | 46,XY,t(1;8;2)(q42;p21;p15) | - | I | classic banding, FISH | A | infertility | [29] |
| 35 | 46,XY,t(9;12;13)(q22;q22;q32) | pat | I | classic banding | AT | MCA | [30] |
| 36 | 46,XY,del(1)(q24.3;q31.1),der(3)(4qter->4q12::1q24.3-> 1q31.1::3p26.2)->3qter),der(4)t(3;4)(p26.2;q12) | - | IV | classic banding, FISH | N | 1 abortion (abnormal) | [31] |
| 37 | 46,XY.t(1;3;6),der(1)(6pter->6p21.2::1p21.1->1qter),  der(3)(3pter->3q22.1::1p22.1->1pter),  der(6)(3qter->3q22.1::1p22.1->1p21.1::6p21.2->6qter) | mat | II | classic banding, FISH | N | Infertility; 1 brother CCR, 1 sister CCR, mother CCR, aunt CCR | [32] |
| 38 | 46,XY,t(1;16)(q21;p11.2),t(8;9)(q24.3;p24) | *de novo*/ mat | III | classic banding | N | infertility; fertil ebrother with t(8;9); fertile cousin with t(8;9) | [33] |
| 39 | 46,XY,t(2;4;9)(p13;q25;p12) | - | I | classic banding | N | 2x RA; 1 healthy son 46,XY | [34] |
| 40 | 46,XY,t(2;4;14)(q21.1;p15.2;q22) | - | I | classic banding | N | 6x RA | [35] |
| 41 | 46,XY,t(3;6)(p24;p21.2),inv(8)(p11;2q21.2 | *de novo* | II | classic banding | N | 3x RA | [36] |
| 42 | 46,XY,t(4;7;15)(q24;q22;q24) | *de novo* | I | classic banding | N | NA | [37] |
| 43 | 46,XY,t(5;11)(p13;q23.2),t(7;14)(q11;q24.1) | - | III | classic banding | N | 4x RA, 1 daughter with cri-du-chat, 1 son with RCT t(7;14) | [38] |
| 44 | 46,XY,der(16)(16pter->16q12::13q14.2->13q14.1:  :5q13->5qter),der(5)(5pter->5q11.2::13q14.2->13qter),  der(13)(13pter->q14.1::16q12->qter) | *de novo* | II | classic banding, FISH | N | RA | [39] |
| 45 | 46,XY,t(1;4;2)(p31.1;q31.3;q24.3) | - | I | classic banding, FISH | NA | 1 healthy child (nonkaryotyped); 2x RA; 1x aborted fetus (abnormal) | [40] |
| 46 | 46,XY,t(1;8)(p31;q21.1),t(8;9)(p23.1;q34) | - | III | classic banding, FISH | NA | 1 miscarriage; 2 non-karyotyped sons; 1 CCR son and 1 CCR daughter | [41] |
| 47 | 46,XY,t(1;12)(p22.1;q22),ins(7;1)(q11.2;q32q42.1) | - | III | classic banding | NA | MCA | [42] |
| 48 | 46,XY,t(2;3;18)(3pter->3p24::2p22->2qter;18qter->18q23: :2p2?4->2p22::3p24->3qter;18pter->18q23::2p2?4->2pter) | - | IV | classic banding, FISH | NA | 2x RA: 1st - CCRpat; 2nd - RCT t(2;18)pat | [43] |
| 49 | 46,XY,der(2)(2pter->2q37.2::6p22.2-> 6p22.2::6p22.1> 6p12.3::2q37.2->2qter),der(6)(6pter->6p22.2::18q21.32-> 18q21.32::6p12.3->6qter),der(18)(18pter->18q21.32: :6p22.2->6p21.1::6p12.3->6p12.3::18q21.32->18qter) | pat | III | classic banding, FISH | NA | MCA in unbalanced CCR son; CCR carriers in family | [44] |
| 50 | 46,t(2;11;22)(q13;q23;q11.22) | mat | I | classic banding | NA | fertile: 3x RA, 1 healthy son 46,XY, 1 daughter with unbalanced karotype | [45, 46] |
| 51 | 46,XY,t(3;4;6)(3pter->3q13::6q13->6qter)(4pter->4q33:  :3q25->3qter)(6qter->6p13::3q12->3q25::4q33->4qter) | - | IV | classic banding | NA | RA, MA balanced | [47] |
| 52 | 46,XY,t(3;7;9)(q23;q22;q22) | - | I | classic banding, FISH | NA | 8x RA, 1 healthy child | [48] |
| 53 | 46,XY,t(3;9)(p11;p23),ins(8;9)(q23;p23) | - | II | classic banding, FISH | NA | 6x RA; 2 daughters with: t(3;9)(p11;p23) or del(9)(p22->ter),ins(8;9)(q23;p23) | [49] |
| 54 | 46,XY,t(3;9)(q10;p10),der(14;15)(q10;q10) | - | III | classic banding, FISH | NA | NA | [50] |
| 55 | 46,XY,der(4)(4pter->4q27::10q11.2->10qter),der(10)  (10pter->10q11.2::14q24.1->14q13::4q28->4qter),  der(14)(14pter->14q13::4q28->4q27::14q24.1->14qter) | *de novo* | III/IV | classic banding, FISH | NA | 1 unbalaced son with t(4;10),+ins(10); 1 healthy son | [51] |
| 56 | 46,XY,t(5;13;8)(q21.2;q14.3;q24.3) | - | I | classic banding, FISH | NA | >2x RA | [52] |
| 57 | 46,XY,t(5;13;16)(q35.1;q12.1;q32.1) | mat | I | classic banding, FISH | NA | 2x RA; 1 daughter der(13) | [50] |
| 58 | 46,XY,t(6;7;10)(q16.2;q34;q26.1) | *de novo* | I | classic banding, FISH | NA | 1 daughter with CCR; 1 son der(10); 2 stillbirths; 1x RA | [53, 54] |
| 59 | 46,XY,t(6;7;18;21)(6pter->6q22::6q25->6qter;7pter->7q21.3 ::21q21.3->21qter;7qter->7q32.1::18p11.21-> 18q21.3: :7q31.3->7q32.1::6q22->6q25::18q21.3->18qter;21pter-> 21q21.3::7q21.3->7q31.3::18p11.21->18pter) | *de novo* | IV | classic banding, FISH | NA | 3 sons with unbalanced CCRs/RCTs: t(6;7;18), t(7;21), t(6;7;18) | [55] |
| 60 | 46,XY,t(6;10;13)(p23;q11;q14) | - | I | classic banding | NA | MCA | [56] |
| 61 | 46,XY,t(6;15)(q16;q21),ins(3;6)(q12;q14q16) | familial | III | classic banding, FISH | NA | MCA | [57] |
| 62 | 46,XY,t(7;9)(q22;p24),ins(8;7)(q21,2;q22q32) | *de novo* | III | classic banding, FISH | NA | 6x RA; 2 abnormal infants perinatally died | [58] |
| 63 | 46,XY,t(9;10;18)(p24;q24;q21) | *de novo* | IV | classic banding | NA | MCA | [59] |
| 64 | 46,XY,t(14;15)(q24.1;q24),t(14';21)(q13;q22.1) | - | II/III | classic banding, FISH | NA | 1 miscarriage; 1 daughter MCA; 1 son CCR (other than father's) | [60] |

References

1. Mahjoubi F, Razazian F. Constitutional complex chromosomal rearrangements in a klinefelter patient: case report and review of literature. J Assist Reprod Genet. 2012 May;29(5):437-441.
2. Bartels I, Starke H, Argyriou L, Sauter SM, Zoll B, Liehr T. An exceptional complex chromosomal rearrangement (CCR) with eight breakpoints involving four chromosomes (1;3;9;14) in an azoospermic male with normal phenotype. Eur J Med Genet. 2007 Mar-Apr;50(2):133-8.
3. Kim JW, Chang EM, Song SH, Park SH, Yoon TK, Shim AH. Complex chromosomal rearrangements in infertile males: complexity of rearrangement affects spermatogenesis. Fertil Steril 2011,95:349-352.
4. Nguyen MH, Morel F, Pennamen P, Parent P, Douet-Guilbert N, Le Bris MJ, Basinko A, Roche S, De Braekeleer M, Perrin A. Balanced complex chromosome rearrangement in male infertility: case report and literature review. Andrologia 2015, 47, 178–185
5. Yakut S, Cetin Z, Clark OA, Usta MF, Berker S, Luleci G. Exceptional complex chromosomal rearrangement and microdeletions at the 4q22.3q23 and 14q31.1q31.3 regions in a patient with azoospermia. Gene. 2013;512:157-160.
6. Salahshourifar I, Gilani MAS, Vosough A, Tavakolzadeh T, Tahsili M, Mansori Z, Karimi H, Totonchi M, Gourabi H. De novo complex chromosomal rearrangement of 46,XY,t(3;16;8)(p26;q13;q21.2) in a non-obstructive azoospermic male. J Appl Genet 47(4), 2006, pp. 93–94
7. Rodriguez MT, Martin MJ, Abrisqueta JA. A complex balanced rearrangement involving four chromosomes in an azoospermic man. J Med Genet. 1985;22:66-7.
8. Wang L, Iqbal F, Li G, Jiang X, Bukhari I, Jiang H, Yang Q, Zhong L, Zhang Y, Hua J, Cooke HJ, Shi Q. Abnormal meiotic recombination with complex chromosomal rearrangement in an azoospermic man. Reprod Biomed Online 2015;30:651-658.
9. Sills ES, Kim JJ, Witt MA, Palermo GD. Non-obstructive azoospermia and maturation arrest with complex translocation 46,XY t(9;13;14)(p22;q21.2;p13) is consistent with the Luciani-Guo hypothesis of latent aberrant autosomal regions and infertility. Cell Chromosome. 2005;14:4:2.
10. Lee IW, Su MT, Hsu CC, Lin YH, Chen PY, Kuo PL. Constitutional Complex Chromosomal Rearrangements In Azoospermic Men – Case Report And Literature Review. Urology 68: 1343.E5–1343.E8, 2006
11. Joseph A, Thomas IM. A complex rearrangement involving three autosomes in a phenotypically normal male presenting with sterility. J Med Genet. 1982,19:375-7.
12. Mouka A, Izard V, Tachdjian G, Brisset S, Yates F, Mayeur A, Drévillon L, Jarray R, Leboulch P, Maouche-Chrétien L, Tosca L. Induced pluripotent stem cell generation from a man carrying a complex chromosomal rearrangement as a genetic model for infertility studies. Sci Rep. 2017 Jan 3;7:39760
13. Coco R, Rahn MI, Estanga PG, Antonioli G, Solari AJ. A constitutional complex chromosome rearrangement involving meiotic arrest in an azoospermic male: case report. Hum Reprod. 2004 Dec;19(12):2784-90
14. Joly-Helas G, de La Rochebrochard C, Mousset-Siméon N, Moirot H, Tiercin C, Romana SP, Le Caignec C, Clavier B, Macé B, Rives N. Complex chromosomal rearrangement and intracytoplasmic sperm injection: a case report. Hum Reprod. 2007 May;22(5):1292-7.
15. Siffroi JP, Benzacken B, Straub B, Le Bourhis C, North MO, Curotti G, Bellec V, Alvarez S, Dadoune JP. Assisted reproductive technology and complex chromosomal rearrangements: the limits of ICSI. Mol Hum Reprod. 1997;3:847-851.
16. Takeshita N, Katagiri Y, Shibui Y, Kitamura M, Fukuda Y, Morita M. Reproductive genetic counseling in patients with complex chromosomal rearrangement. Fertil Steril 2007;88:S240-S241.
17. Ergul E, Liehr T, Mrasek K, Sazci A. 2009 A de novo complex chromosome rearrangement involving three chromosomes (2, 13, and 18) in an oligozoospermic male. Fertility and Sterility 92 391.e9–391.e12
18. Pellestor F, Puechberty J, Weise A, Lefort G, Anahory T, Liehr T & Sarda P 2011b Meiotic segregation of complex reciprocal translocations: direct analysis of the spermatozoa of a t(5;13;14) carrier. Fertility and Sterility 95 e17–e22
19. Li L, Heng X, Yun W, Zheng S, Zhang J, Fan W. Familial complex chromosome rearrangement (CCR) involving 5 breakpoints on chromosomes 1, 3 and 13 in a severe oligozoospermic patient. J Assist Reprod Genet. 2013;30:423-9.
20. Loup V, Bernicot I, Janssens P, Hedon B, Hamamah S, Pellestor F & Anahory T 2010 Combined FISH and PRINS sperm analysis of complex chromosome rearrangement t(1;19;13): an approach facilitating PGD. Molecular Human Reproduction 16 111–116
21. Chen Y-J, Zhang W-W, Sun X-M, Hu C-J. A rare complex chromosomal rearrangement in an oligozoospermic male: a case report and review of the Chinese literature. Asian J Androl, 2014, 16:325-326
22. Ferfouri F, Boitrelle F, Clement P, Molina Gomes D, Selva J & Vialard F 2014 Sperm FISH analysis of a 44,X,der(Y),t(Y;15)(q12;q10)pat, rob (13;14)(q10;q10)mat complex chromosome rearrangement. Andrologia. In press
23. Kirkpatrick G, Ma S 2012 Meiotic segregation and interchromosomal effects in a rare (1:2:10) complex chromosomal rearrangement. J Assist Reprod Genet 29 77–81
24. Salahshourifar I, Shahrokhshahi N, Tavakolzadeh T, Beheshti Z, Gourabi H. Complex chromosomal rearrangement involving chromosomes 1, 4 and 22 in an infertile male: case report and literature review. J Appl Genet 50(1), 2009, pp. 69–72
25. Olszewska M, Huleyuk N, Fraczek M, Zastavna D, Wiland E, Kurpisz M. Sperm FISH and chromatin integrity in spermatozoa from a t(6;10;11) carrier. Reproduction (2014) 147 659–670
26. Lebbar A, Callier P, Baverel F, Marle N, Patrat C, Le Tessier D, Mugneret F, Dupont JM. Two cases of mosaicism for complex chromosome rearrangements (CCRM) associated with secondary infertility. Am J Med Genet A. 2008 Oct 15;146A(20):2651-6
27. Pierron L, Irrmann A, de Chalus A, Bloch A, Heide S, Rogers E, Ledee N, Prat-Ellenberg L, Coussement A, Dupont J-M, Cassuto NG, Siffroi J-P, Rouen A. Double chromosomal translocation in an infertile man: one-step FISH meiotic segregation analysis and reproductive prognosis. J Assist Reprod Genet 36: 973-978
28. Asia S, Vaziri Nasab H, Sabbaghian M, Kalantari H, Moradi SZ, Gourabi H, Meybodi AM. A Rare De novo Complex Chromosomal Rearrangement (CCR) Involving Four Chromosomes in An Oligo-asthenosperm Infertile Man. Cell J. 2014 Fall;16(3):377-82
29. Godo A, Blanco J, Vidal F, Parriego M, Boada M & Anton E 2013 Sequential FISH allows the determination of the segregation outcome and the presence of numerical anomalies in spermatozoa from a t(1;8;2) (q42;p21;p15) carrier. Journal of Assisted Reproduction and Genetics 30 1115–1123
30. Johannisson R, Lohrs U, Passarge E. 1988 Pachytene analysis in males heterozygous for a familial translocation (9;12;13)(q22;q22;q32) ascertained through a child with partial trisomy 9. Cytogenetics and Cell Genetics 47 160–166
31. Priya PK, Mishra VV, Liehr T, Ziegler M, Tiwari S, Patel A, Chettiar SS, Patel H. Characterization of a complex chromosomal rearrangement involving chromosomes 1, 3, and 4 in a slightly affected male with bad obstetrics history. J Assist Reprod Genet. 2018 Apr;35(4):721-725
32. Hornak M, Vozdova M, Musilova P, Prinosilova P, Oracova E, Linkova V, Vesela K, Rubes J. Comprehensive meiotic segregation analysis of a 4-breakpoint t(1;3;6) complex chromosome rearrangement using single sperm array comparative genomic hybridization and FISH. Reproductive BioMedicine Online (2014) 29, 499–508
33. Ferfouri F, Boitrelle F, Clement P, Gomes DM, Selva J & Vialard F 2013 Can one translocation impact the meiotic segregation of another translocation? A sperm-FISH analysis of a 46,XY,t(1;16)(q21;p11.2), t(8;9)(q24.3;p24) patient and his 46,XY,t(8;9)(q24.3;p24) brother and cousin. Mol Hum Reprod 19 109–117
34. Saadallah N, Hulten M 1985 A complex three breakpoint translocation involving chromosomes 2, 4, and 9 identified by meiotic investigations of a human male ascertained for subfertility. Human Genetics 71 312–320
35. Mas J, Sabouni R, Bocca S. A novel male 2;4;14 complex chromosomal translocation with normal semen parameters but 100% embryonic aneuploidy. Journal of Assisted Reproduction and Genetics (2018) 35:907–912
36. Ferfouri F, Biotrelle F, Tapia S, Gomes DM, Selva J & Vialard F 2012 Sperm FISH analysis of a 46,XY,t(3;6)(p24;p21.2), inv(8)(p11;2q21.2) double chromosomal rearrangement. Reproductive Biomedicine Online 24 219–223
37. Chandley AC, Edmondt P, Christie S, Gowans L, Fletcher J, Frackiewicz A, Newton M. Cytogenetics and infertility in man. Ann. Hum. Genet., Lot Ed. (1975), 39, 231-254
38. Burns JP, Koduru PRK, Alonso ML & Chaganti RSK 1986 Analysis of meiotic segregation in a man heterozygous for two reciprocal translocations using the hamster in vitro penetration system. American Journal of Human Genetics 38 954–964
39. Farra C, Singer S, Dufke A, Ashkar H, Monsef C, Awwad J. De novo exceptional complex chromosomal rearrangement in a healthy fertile male: case report and review of the literature. Fertil Steril. 2011 96(5):1160-4.
40. Karadeniz N, Mrasek K, Weise A. Further delineation of complex chromosomal rearrangements in fertile male using multicolor banding. Mol Cytogenet. 2008 Aug 7;1:17
41. Zahed L, Der Kaloustian V, Batanian JR. Familial Complex Chromosome Rearrangement Giving Rise to Balanced and Unbalanced Recombination Products. American Journal of Medical Genetics 79:30–34 (1998)
42. Schmidt A, Passarge E. A complex familial translocation with a five-break rearrangement involving three chromosomes. Clin Genet 1988;34(Suppl):415
43. Basinko A, Perrin A, Nguyen HA, Morel F, Le Bris M-J, Saliou A-H, Collet M, Parent P, Benech C, Quemener S et al. 2010 Balanced transmission of a paternal complex chromosomal rearrangement involving chromosomes 2, 3, and 18. Am J Med Genet 152A 2646–2650
44. Gruchy N, Barreau M, Kessler K, Gourdier D & Leporrier N 2010 A paternally transmitted complex chromosomal rearrangement (CCR) involving chromosomes 2, 6, and 18 includes eight breakpoints and five insertional translocations (ITs) through three generations. American Journal of Medical Genetics 152A 185–190
45. Cifuentes P, Navarro J, Miguez L, Egozcue J, Benet J. 1998 Sperm segregation analysis of a complex chromosome rearrangement, 2;22;11, by whole chromosome painting. Cytogenetics and Cell Genetics 82 204–209
46. Fuster C, Miguez L, Miró R, Rigola MA, Perez A, Egozcue J. Familial complex chromosome rearrangement ascertained by in situ hybridisation. J Med Genet. 1997 Feb;34(2):164-6
47. Gorski JL, Kistenmacher MN, Punnett HH, Zackai EH & Emanuel BS 1988 Reproductive risks for carriers of complex chromosome rearrangements: analysis of 25 families. American Journal of Medical Genetics 29A 247–261
48. Frumkin T, Peleg S, Gold V, Reches A, Asaf S, Azem F, Ben-Yosef D, Malcov M. Complex chromosomal rearrangement – a lesson learned from PGS. J Assist Reprod Genet (2017) 34:1095–1100
49. Wagstaff J, Hemann M. A familial "balanced" 3;9 translocation with cryptic 8q insertion leading to deletion and duplication of 9p23 loci in siblings. Am J Hum Genet. 1995;56:302-309.
50. Escudero T, Estop A, Fischer J & Munne S 2008 Preimplantation genetic diagnosis for complex chromosome rearrangements. American Journal of Medical Genetics 146A 1662–1669
51. Grasshoff U, Singer S, Liehr T, Starke H, Fode B, Schoenig M & Dufke A 2003 A complex chromosomal rearrangement with a translocation 4;10;14 in a fertile carrier: ascertainment through an offspring with partial trisomy 14q13/q21.1 and partial monosomy 4q27/q28. Cytogenetic and Genome Research 103 17–23
52. Lim CK, Cho JW, Kim JY, Kang IS, Shim SH & Jun JH 2008 A healthy live birth after successful preimplantation diagnosis for carriers of complex chromosome rearrangements. Fertility and Sterility 90 1680–1684
53. Patsalis PC, Evangelidou P, Charalambous S, Sismani C. Fluorescence in situ hybridization characterization of apparently balanced translocation reveals cryptic complex chromosomal rearrangements with unexpected level of complexity. Eur J Hum Genet. 2004 Aug;12(8):647-53
54. Aristidou C, Theodosiou A, Bak M, Mehrjouy MM, Constantinou E, Alexandrou A, Papaevripidou I, Christophidou-Anastasiadou V, Skordis N, Kitsiou-Tzeli S, Tommerup N, Sismani C. Position effect, cryptic complexity, and direct gene disruption as disease mechanisms in de novo apparently balanced translocation cases. PLoS One. 2018 Oct 5;13(10):e0205298
55. Röthlisberger B, Kotzot D, Brecevic L, Koehler M, Balmer D, Binkert F, Schinzel A. Recombinant balanced and unbalanced translocations as a consequence of a balanced complex chromosomal rearrangement involving eight breakpoints in four chromosomes. Eur J Hum Genet. 1999;7:873-83.
56. Schwanitz G, Schmid P, Berthold HJ, Grosse KP. Partial trisomy 13 with clinical signs of Patau syndrome, resulting from a complex paternal rearrangement of chromosomes 6, 10 and 13. Ann Genet 1978;21:100-103.
57. Roland B, Lowry RB, Cox DM, Ferreira P, Lin CC. Familial complex chromosomal rearrangement resulting in duplication/deletion of 6q14 to 6q16. Clin Genet 1993;43:117-121.
58. Cai T, Yu P, Tagle DA, Lu D, ChenY, Xia J. 2001 A de novo complex chromosomal rearrangement with a translocation 7;9 and 8q insertion in a male carrier with no infertility. Human Reproduction 16 59–62
59. Bourrouillou G, Rolland M, Colombies P. Secondary 18q2 due to a paternal double translocation. J Genet Hum. 1983 31(3):243-9
60. 87 Soler A, Sanchez A, Carrio A, Badenas C, Mila M, Margarit E & Borrell A. 2005 Recombination in a male carrier of two reciprocal translocations involving chromosomes 14, 140, 15, and 21 leading to balanced and unbalanced rearrangements in offspring. American Journal of Medical Genetics 134A 309–314
