# Supplementary material for: Familial Infertility (Azoospermia and Cryptozoospermia) in Two Brothers—Carriers of t(1;7) Complex Chromosomal Rearrangement (CCR): Molecular Cytogenetic Analysis
Source: Int J Mol Sci. 2020 Jun 26;21(12):4559. doi: 10.3390/ijms21124559 (PMC7349667; doi:10.3390/ijms21124559)
Supplement: Supplementary file 1 [file ijms-21-04559-s001.zip › Supplementary Table2.docx]

| **Chromosome** | **Band** | **Start** | **Stop** | **Probes** | **Amplification** | **Deletion** | **P value** | **Genes** | **Sample** | **Maternal inheritance** | **In both members** | **Function notes** |
| --- | --- | --- | --- | --- | --- | --- | --- | --- | --- | --- | --- | --- |
| chr2 | q31.2 | 180 069 605 | 180 070 733 | 3 | 0,784413 | 0 | 2,63E-10 | *SESTD1* | Mother | Yes | Shared | SEC14 and spectrin domain containing 1, no fertility function |
| chr2 | q31.2 | 180 069 605 | 180 070 733 | 3 | 1,009107 | 0 | 7,41E-15 | *SESTD1* | Patient | Yes | Shared | SEC14 and spectrin domain containing 1, no fertility function |
| chr6 | q27 | 165 725 547 | 165 737 665 | 3 | 0,954852 | 0 | 1,47E-13 |  | Patient | No | Not Shared | No genes |
| chr9 | q33.3 | 130 041 553 | 130 145 721 | 23 | 0 | -0,97556 | NA | *GARNL3* | Patient | No | Not Shared | GTPase activating Rap/RanGAP domain-like 3, no fertility function |
| chr11 | p14.3 | 25 635 357 | 25 764 082 | 9 | 0,605224 | 0 | 3,80E-17 |  | Mother | Yes | Shared | No genes, testis-specific expression in RP11-405K6.1, non-coding RNA |
| chr11 | p14.3 | 25 635 357 | 25 764 082 | 9 | 0,69559 | 0 | 1,35E-22 |  | Patient | Yes | Shared | No genes, testis-specific expression in RP11-405K6.1, non-coding RNA |
| chrX | p22.33 | 1 731 610 | 1 752 284 | 10 | 0,519465 | 0 | 3,75E-15 | *ASMT* | Patient | No | Not Shared | acetylserotonin O-methyltransferase, melatonin synthesis |
| chrY | p11.32 | 1 681 610 | 1 702 284 | 10 | 0,519465 | 0 | 3,75E-15 | *ASMT* | Patient | No | Not Shared | acetylserotonin O-methyltransferase, melatonin synthesis |

**Supplementary Table S2** Non-polymorphic variants found in aCGH screening of brother-1 and mother samples.
