# Supplementary material for: Familial Infertility (Azoospermia and Cryptozoospermia) in Two Brothers—Carriers of t(1;7) Complex Chromosomal Rearrangement (CCR): Molecular Cytogenetic Analysis
Source: Int J Mol Sci. 2020 Jun 26;21(12):4559. doi: 10.3390/ijms21124559 (PMC7349667; doi:10.3390/ijms21124559)
Supplement: Supplementary file 1 [file ijms-21-04559-s001.zip › Supplementary Table3.docx]

**Supplementary Table S3** Final result of aCGH screening – list of genes found, incl. their role. Three known nonpolymorphic genes, *ASMT*, *GARNL3,* *SESTD1,* were ruled out due to unlikely biological function and nonspecific testis tissue expression profile.

| # | **Gene** | **Source** | **Maternal inheritance** | **MGI** | **BioGPS** | **Unigene** | **NCBI notes** | **Other notes** |
| --- | --- | --- | --- | --- | --- | --- | --- | --- |
| 1 | *AVL9* | Single probe | Yes | N | 0 | 1 | cell migration associated |  |
| 2 | *BBS9* | Single probe | Yes | N | 1 | 1 | This gene is downregulated by parathyroid hormone in osteoblastic cells, and therefore is thought to be involved in parathyroid hormone action in bones. The exact function of this gene has not yet been determined. |  |
| 3 | *BMPER* | Single probe | Yes | 0 | 0 | 0 | This gene encodes a secreted protein that interacts with, and inhibits bone morphogenetic protein (BMP) function. It has been shown to inhibit BMP2- and BMP4-dependent osteoblast differentiation and BMP-dependent differentiation of the chondrogenic cells. Mutations in this gene are associated with a lethal skeletal disorder, diaphanospondylodysostosis. |  |
| 4 | *CCDC76* | Single probe | Yes | N | 1 | 1 | None |  |
| 5 | *COL11A1* | Single probe | Yes | 0 | 0 | 1 | This gene encodes one of the two alpha chains of type XI collagen, a minor fibrillar collagen. Type XI collagen is a heterotrimer but the third alpha chain is a post-translationally modified alpha 1 type II chain. Mutations in this gene are associated with type II Stickler syndrome and with Marshall syndrome. A single-nucleotide polymorphism in this gene is also associated with susceptibility to lumbar disc herniation. Multiple transcript variants have been identified for this gene. [provided by RefSeq, Nov 2009] |  |
| 6 | *DPY19L1* | Single probe | Yes | N | 2 | 1 | None |  |
| 7 | *DPYD* | Single probe | Yes | 1 | 1 | 1 | The protein encoded by this gene is a pyrimidine catabolic enzyme and the initial and rate-limiting factor in the pathway of uracil and thymidine catabolism. Mutations in this gene result in dihydropyrimidine dehydrogenase deficiency, an error in pyrimidine metabolism associated with thymine-uraciluria and an increased risk of toxicity in cancer patients receiving 5-fluorouracil chemotherapy. Two transcript variants encoding different isoforms have been found for this gene. [provided by RefSeq, May 2009] | MGI abnormal epididymis morphology |
| 8 | *GHRHR* | Single probe | Yes | 1 | 0 | 0 | This gene encodes a receptor for growth hormone-releasing hormone. Binding of this hormone to the receptor leads to synthesis and release of growth hormone. Mutations in this gene have been associated with isolated growth hormone deficiency (IGHD), also known as Dwarfism of Sindh, a disorder characterized by short stature. [provided by RefSeq, Jun 2010] | MGI reduced male fertility, hormonal abnormalities |
| 9 | *GPSM2* | Single probe | Yes | 0 | 1 | 1 | The protein encoded by this gene belongs to a family of proteins that modulate activation of G proteins, which transduce extracellular signals received by cell surface receptors into integrated cellular responses. The N-terminal half of this protein contains 10 copies of leu-gly-asn (LGN) repeat, and the C-terminal half contains 4 GoLoco motifs, which are involved in guanine nucleotide exchange. This protein may play a role in neuroblast division and in the development of normal hearing. Mutations in this gene are associated with autosomal recessive nonsyndromic deafness (DFNB82). Alternative splicing results in multiple transcript variants. [provided by RefSeq, Mar 2016] |  |
| 10 | *LOC100129620* | Single probe | Yes | N | 1 | 0 | None |  |
| 11 | *LOC729970* | Single probe | Yes | N | 2 | 1 | None |  |
| 12 | *LPPR5* | Single probe | Yes | N | 1 | 0 | The protein encoded by this gene is a type 2 member of the phosphatidic acid phosphatase (PAP) family. All type 2 members of this protein family contain 6 transmembrane regions, and a consensus N-glycosylation site. PAPs convert phosphatidic acid to diacylglycerol, and function in de novo synthesis of glycerolipids as well as in receptor-activated signal transduction mediated by phospholipase D. Alternate transcriptional splice variants, encoding different isoforms, have been characterized. [provided by RefSeq, Jul 2008] |  |
| 13 | *MIR548AA1* | Single probe | Yes | N | N | N | microRNAs (miRNAs) are short (20-24 nt) non-coding RNAs that are involved in post-transcriptional regulation of gene expression in multicellular organisms by affecting both the stability and translation of mRNAs. miRNAs are transcribed by RNA polymerase II as part of capped and polyadenylated primary transcripts (pri-miRNAs) that can be either protein-coding or non-coding. The primary transcript is cleaved by the Drosha ribonuclease III enzyme to produce an approximately 70-nt stem-loop precursor miRNA (pre-miRNA), which is further cleaved by the cytoplasmic Dicer ribonuclease to generate the mature miRNA and antisense miRNA star (miRNA*) products. The mature miRNA is incorporated into a RNA-induced silencing complex (RISC), which recognizes target mRNAs through imperfect base pairing with the miRNA and most commonly results in translational inhibition or destabilization of the target mRNA. The RefSeq represents the predicted microRNA stem-loop. [provided by RefSeq, Sep 2009] |  |
| 14 | *MIR548D1* | Single probe | Yes | N | N | N | microRNAs (miRNAs) are short (20-24 nt) non-coding RNAs that are involved in post-transcriptional regulation of gene expression in multicellular organisms by affecting both the stability and translation of mRNAs. miRNAs are transcribed by RNA polymerase II as part of capped and polyadenylated primary transcripts (pri-miRNAs) that can be either protein-coding or non-coding. The primary transcript is cleaved by the Drosha ribonuclease III enzyme to produce an approximately 70-nt stem-loop precursor miRNA (pre-miRNA), which is further cleaved by the cytoplasmic Dicer ribonuclease to generate the mature miRNA and antisense miRNA star (miRNA*) products. The mature miRNA is incorporated into a RNA-induced silencing complex (RISC), which recognizes target mRNAs through imperfect base pairing with the miRNA and most commonly results in translational inhibition or destabilization of the target mRNA. The RefSeq represents the predicted microRNA stem-loop. [provided by RefSeq, Sep 2009] |  |
| 15 | *PALMD* | Single probe | Yes | N | 1 | 1 | None, literature about apoptosis in response to DNA damage |  |
| 16 | *PCNXL2* | Single probe | Yes | N | 1 | 1 | This gene contains coding mononucleotide repeats that are associated with tumors of high mcrosatellite instability (MSI-H). Defects in this gene are involved in the tumorigenesis of MSI-H colorectal carcinomas. [provided by RefSeq, Jun 201 |  |
| 17 | *PDE1C* | Single probe | Yes | 0 | 1 | 1 | This gene encodes an enzyme that belongs to the 3'5'-cyclic nucleotide phosphodiesterase family. Members of this family catalyze hydrolysis of the cyclic nucleotides, cyclic adenosine monophosphate and cyclic guanosine monophosphate, to the corresponding nucleoside 5'-monophosphates. The enzyme encoded by this gene regulates proliferation and migration of vascular smooth muscle cells, and neointimal hyperplasia. This enzyme also plays a role in pathological vascular remodeling by regulating the stability of growth factor receptors, such as PDGF-receptor-beta. [provided by RefSeq, Jul 2016] |  |
| 18 | *PTBP2* | Single probe | Yes | 0 | 1 | 2 | The protein encoded by this gene binds to intronic polypyrimidine clusters in pre-mRNA molecules and is implicated in controlling the assembly of other splicing-regulatory proteins. This protein is very similar to the polypyrimidine tract binding protein (PTB) but most of its isoforms are expressed primarily in the brain. Alternative splicing results in multiple transcript variants. [provided by RefSeq, Jul 2014] |  |
| 19 | *SLC35A3* | Single probe | Yes | N | 0 | 1 | This gene encodes a UDP-N-acetylglucosamine transporter found in the golgi apparatus membrane. In cattle, a missense mutation in this gene causes complex vertebral malformation. Alternative splicing results in multiple transcript variants. [provided by RefSeq, Nov 2012] |  |
| 20 | *SLC35F3* | Single probe | Yes | N | 0 | 1 | None |  |
| 21 | *SNX7* | Single probe | Yes | N | 1 | 1 | This gene encodes a member of the sorting nexin family. Members of this family contain a phox (PX) domain, which is a phosphoinositide binding domain, and are involved in intracellular trafficking. This protein does not contain a coiled coil region like some family members, and its exact function is unknown. Alternative splicing results in multiple transcript variants. A related pseudogene has been identified on chromosome 11. [provided by RefSeq, Jun 2010] |  |
| 22 | *TARBP1* | Single probe | Yes | N | 0 | 1 | HIV-1, the causative agent of acquired immunodeficiency syndrome (AIDS), contains an RNA genome that produces a chromosomally integrated DNA during the replicative cycle. Activation of HIV-1 gene expression by the transactivator Tat is dependent on an RNA regulatory element (TAR) located downstream of the transcription initiation site. This element forms a stable stem-loop structure and can be bound by either the protein encoded by this gene or by RNA polymerase II. This protein may act to disengage RNA polymerase II from TAR during transcriptional elongation. Alternatively spliced transcripts of this gene may exist, but their full-length natures have not been determined. [provided by RefSeq, Jul 2008] |  |
| 23 | *VAV3* | Single probe | Yes | N | 1 | 1 | This gene is a member of the VAV gene family. The VAV proteins are guanine nucleotide exchange factors (GEFs) for Rho family GTPases that activate pathways leading to actin cytoskeletal rearrangements and transcriptional alterations. This gene product acts as a GEF preferentially for RhoG, RhoA, and to a lesser extent, RAC1, and it associates maximally with the nucleotide-free states of these GTPases. Alternatively spliced transcript variants encoding different isoforms have been described for this gene. [provided by RefSeq, Jul 2008] |  |
| 24 | ***SESTD1*** | ADM-2 variant | Yes | 0 | 1 | 1 | SEC14 and spectrin domain containing 1, no fertility function | Rare variant |
| 25 | ***GARNL3*** | ADM-2 variant | No | N | 0 | 1 | GTPase activating Rap/RanGAP domain-like 3, no fertility function | Rare variant |
| 26 | ***ASMT*** | ADM-2 variant | No | N | 1 | 1 | This gene belongs to the methyltransferase superfamily, and is located in the pseudoautosomal region (PAR) at the end of the short arms of the X and Y chromosomes. The encoded enzyme catalyzes the final reaction in the synthesis of melatonin, and is abundant in the pineal gland. Alternatively spliced transcript variants have been noted for this gene. [provided by RefSeq, Jan 2010] | Rare variant |
