# Supplementary material for: Familial Infertility (Azoospermia and Cryptozoospermia) in Two Brothers—Carriers of t(1;7) Complex Chromosomal Rearrangement (CCR): Molecular Cytogenetic Analysis
Source: Int J Mol Sci. 2020 Jun 26;21(12):4559. doi: 10.3390/ijms21124559 (PMC7349667; doi:10.3390/ijms21124559)
Supplement: Supplementary file 1 [file ijms-21-04559-s001.zip › Supplementary Table4.docx]

| **Chromosome** | **Band** | **Start (bp)** | **Stop (bp)** | **Size (bp)** | **CGH Probes** | **Average probe interval (bp)** |
| --- | --- | --- | --- | --- | --- | --- |
| 1 | 1p21.3 | 95 000 000 | 110 000 000 | 15 000 000 | 1830 | 8196,721311 |
| 1 | 1q42.3 | 233 000 000 | 235 000 000 | 2 000 000 | 323 | 6191,950464 |
| 7 | 7p14.3 | 31 000 000 | 35 000 000 | 4 000 000 | 651 | 6144,393241 |

**Supplementary Table S4** Coordinates of 3 breakpoint regions of the translocation analyzed using a higher resolution analysis with individual aCGH probes. The total number of probes used was 2804.
