# Supplementary material for: Familial Infertility (Azoospermia and Cryptozoospermia) in Two Brothers—Carriers of t(1;7) Complex Chromosomal Rearrangement (CCR): Molecular Cytogenetic Analysis
Source: Int J Mol Sci. 2020 Jun 26;21(12):4559. doi: 10.3390/ijms21124559 (PMC7349667; doi:10.3390/ijms21124559)
Supplement: Supplementary file 1 [file ijms-21-04559-s001.zip › Supplementary Table5.docx]

**Supplementary Table S5** aCGH results obtained for 3 breakpoint regions of the translocation.

| AberrationNo | Chr | Cytoband | Start | Stop | #Probes | Amplification | Gain | Loss | Deletion | P value | Gene Names | Maternal inhertance |
| --- | --- | --- | --- | --- | --- | --- | --- | --- | --- | --- | --- | --- |
| 2 | chr1 | p21.3 | 95 158 598 | 95 158 657 | 1 | 0 | 0,312634 | 0 | 0 | 0,08404 |  | No |
| 3 | chr1 | p21.3 | 95 236 830 | 95 236 889 | 1 | 0 | 0 | -0,39296 | 0 | 0,03604 |  | No |
| 5 | chr1 | p21.3 | 95 658 446 | 95 658 505 | 1 | 0 | 0,393375 | 0 | 0 | 0,03514 | *TMEM56, TMEM56-RWDD3* | No |
| 7 | chr1 | p21.3 | 95 958 347 | 95 958 406 | 1 | 0 | 0 | -0,34011 | 0 | 0,06416 |  | No |
| 8 | chr1 | p21.3 | 96 906 118 | 96 906 177 | 1 | 0 | 0 | -0,31396 | 0 | 0,2979 |  | No |
| 10 | chr1 | p21.3 | 97 227 002 | 97 227 061 | 1 | 0 | 0,337856 | 0 | 0 | 0,06475 | *PTBP2* | No |
| 12 | chr1 | p21.3 | 97 375 832 | 97 375 891 | 1 | 0 | 0 | -0,35655 | 0 | 0,4792 |  | No |
| 13 | chr1 | p21.3 | 97 602 497 | 97 602 556 | 1 | 0 | 0 | -0,31434 | 0 | 0,08263 | *DPYD* | No |
| 14 | chr1 | p21.3 | 97 862 419 | 97 862 478 | 1 | 0 | 0,338772 | 0 | 0 | 0,06478 | *DPYD* | No |
| 18 | chr1 | p21.3 | 98 093 127 | 98 093 186 | 1 | 0 | 0,443002 | 0 | 0 | 0,4714 | *DPYD* | No |
| 17 | chr1 | p21.3 | 98 093 127 | 98 100 486 | 3 | 0 | 0,302175 | 0 | 0 | 0,006502 | *DPYD* | No |
| 20 | chr1 | p21.3 | 98 672 966 | 98 673 025 | 1 | 0 | 0,325773 | 0 | 0 | 0,07398 |  | No |
| 21 | chr1 | p21.3 | 98 705 153 | 98 705 212 | 1 | 0 | 0 | -0,35409 | 0 | 0,0583 |  | No |
| 22 | chr1 | p21.3 | 98 746 393 | 98 746 452 | 1 | 0 | 0 | -0,31281 | 0 | 0,08554 |  | No |
| 28 | chr1 | p21.2 | 99 969 148 | 99 969 207 | 1 | 0 | 0 | -0,46761 | 0 | 0,01532 |  | No |
| 29 | chr1 | p21.2 | 100 127 855 | 100 127 914 | 1 | 0 | 0 | -0,32556 | 0 | 0,07368 | *PALMD* | No |
| 31 | chr1 | p21.2 | 100 201 414 | 100 201 473 | 1 | 0 | 0,315248 | 0 | 0 | 0,08448 | *FRRS1* | No |
| 32 | chr1 | p21.2 | 100 283 708 | 100 283 767 | 1 | 0 | 0,315476 | 0 | 0 | 0,08139 |  | No |
| 35 | chr1 | p21.2 | 100 424 650 | 100 424 709 | 1 | 0 | 0,373811 | 0 | 0 | 0,04457 |  | No |
| 38 | chr1 | p21.2 | 101 105 605 | 101 105 664 | 1 | 0 | 0,335706 | 0 | 0 | 0,06592 |  | No |
| 39 | chr1 | p21.2 | 101 409 923 | 101 409 982 | 1 | 0 | 0 | -0,55362 | 0 | 0,005847 | *SLC30A7* | No |
| 40 | chr1 | p21.2 | 101 422 421 | 101 422 480 | 1 | 0 | 0,324529 | 0 | 0 | 2,238 | *SLC30A7* | No |
| 41 | chr1 | p21.2 | 101 487 256 | 101 487 315 | 1 | 0 | 0 | -0,3298 | 0 | 0,07003 | *DPH5* | No |
| 42 | chr1 | p21.2 | 101 588 324 | 101 588 383 | 1 | 0 | 0,372265 | 0 | 0 | 0,4257 |  | No |
| 43 | chr1 | p21.2 | 101 713 048 | 101 713 107 | 1 | 0 | 0 | -0,35147 | 0 | 0,05775 |  | No |
| 44 | chr1 | p21.2 | 101 724 299 | 101 724 358 | 1 | 0 | 0,34704 | 0 | 0 | 0,4922 |  | No |
| 45 | chr1 | p21.2 | 101 820 067 | 101 820 126 | 1 | 0 | 0 | -0,33164 | 0 | 1,148 |  | No |
| 46 | chr1 | p21.2 | 101 894 729 | 101 894 788 | 1 | 0 | 0,325718 | 0 | 0 | 0,07337 |  | No |
| 47 | chr1 | p21.1 | 102 326 183 | 102 326 242 | 1 | 0 | 0 | -0,92313 | 0 | 1,93E-05 | *OLFM3* | No |
| 48 | chr1 | p21.1 | 102 411 931 | 102 411 990 | 1 | 0 | 0,304378 | 0 | 0 | 0,27 | *OLFM3* | No |
| 49 | chr1 | p21.1 | 102 656 654 | 102 677 365 | 2 | 0 | 0,307286 | 0 | 0 | 1,757 |  | No |
| 51 | chr1 | p21.1 | 103 129 971 | 103 130 030 | 1 | 0 | 0,322691 | 0 | 0 | 0,07565 |  | No |
| 53 | chr1 | p21.1 | 103 339 946 | 103 340 005 | 1 | 0 | 0 | -0,38369 | 0 | 1,325 |  | No |
| 52 | chr1 | p21.1 | 103 339 946 | 103 343 344 | 2 | 0 | 0 | -0,32293 | 0 | 0,01661 | *COL11A1* | No |
| 56 | chr1 | p21.1 | 104 115 055 | 104 115 114 | 1 | 0 | 0 | -0,53864 | 0 | 0,006556 | *AMY2B* | No |
| 58 | chr1 | p21.1 | 104 353 374 | 104 353 433 | 1 | 0 | 0,346331 | 0 | 0 | 0,06139 |  | No |
| 59 | chr1 | p21.1 | 104 456 370 | 104 456 429 | 1 | 0 | 0 | -0,42103 | 0 | 0,02591 |  | No |
| 60 | chr1 | p21.1 | 104 570 446 | 104 570 505 | 1 | 0 | 0,312611 | 0 | 0 | 0,2976 |  | No |
| 62 | chr1 | p21.1 | 104 601 724 | 104 601 783 | 1 | 0 | 0 | -0,37706 | 0 | 1,112 |  | No |
| 61 | chr1 | p21.1 | 104 601 724 | 104 613 985 | 3 | 0 | 0 | -0,30581 | 0 | 0,006008 |  | No |
| 65 | chr1 | p21.1 | 105 869 766 | 105 869 825 | 1 | 0 | 0,348677 | 0 | 0 | 0,5472 |  | No |
| 66 | chr1 | p21.1 | 105 972 950 | 105 973 009 | 1 | 0 | 0,312214 | 0 | 0 | 0,08473 |  | No |
| 70 | chr1 | p21.1 | 106 769 435 | 106 769 494 | 1 | 0 | 0 | -0,3108 | 0 | 0,08791 |  | No |
| 72 | chr1 | p13.3 | 107 823 240 | 107 823 299 | 1 | 0 | 0,368326 | 0 | 0 | 0,159 | *NTNG1* | No |
| 73 | chr1 | p13.3 | 107 840 958 | 107 841 017 | 1 | 0 | 0,36 | 0 | 0 | 0,1731 | *NTNG1* | No |
| 74 | chr1 | p13.3 | 107 938 073 | 107 938 132 | 1 | 0 | 0,312224 | 0 | 0 | 0,1538 | *NTNG1* | No |
| 75 | chr1 | p13.3 | 108 029 776 | 108 029 835 | 1 | 0 | 0,303708 | 0 | 0 | 0,1674 |  | No |
| 76 | chr1 | p13.3 | 108 111 006 | 108 111 065 | 1 | 0 | 0 | -0,48381 | 0 | 0,0124 |  | No |
| 78 | chr1 | p13.3 | 108 540 547 | 108 540 606 | 1 | 0 | 0,316718 | 0 | 0 | 0,08028 |  | No |
| 79 | chr1 | p13.3 | 108 661 659 | 108 661 718 | 1 | 0 | 0,368006 | 0 | 0 | 0,04703 |  | No |
| 80 | chr1 | p13.3 | 108 724 558 | 108 724 614 | 1 | 0 | 0 | -0,33161 | 0 | 0,08865 | *SLC25A24* | No |
| 84 | chr1 | p13.3 | 109 203 098 | 109 203 157 | 1 | 0 | 0,360169 | 0 | 0 | 0,1231 | *HENMT1* | No |
| 85 | chr1 | p13.3 | 109 450 510 | 109 450 569 | 1 | 0 | 0,375799 | 0 | 0 | 0,04474 | *GPSM2* | No |
| 87 | chr1 | p13.3 | 109 840 880 | 109 840 938 | 1 | 0 | 0,360517 | 0 | 0 | 0,05067 | *MYBPHL* | No |
| 88 | chr1 | p13.3 | 109 884 743 | 109 884 802 | 1 | 0 | 0,502392 | 0 | 0 | 0,01085 | *SORT1* | No |
| 92 | chr1 | q42.2 | 233 237 053 | 233 237 112 | 1 | 0 | 0 | -0,6008 | 0 | 0,003668 | *PCNXL2* | No |
| 94 | chr1 | q42.2 | 233 436 766 | 233 436 825 | 1 | 0 | 0 | -0,56916 | 0 | 0,004283 |  | No |
| 96 | chr1 | q42.2 | 233 955 333 | 233 955 392 | 1 | 0 | 0,389806 | 0 | 0 | 0,03653 |  | No |
| 97 | chr1 | q42.2 | 234 039 835 | 234 039 894 | 1 | 0 | 0 | -0,43201 | 0 | 0,02355 |  | No |
| 98 | chr1 | q42.2 | 234 253 955 | 234 254 014 | 1 | 0 | 0,504819 | 0 | 0 | 0,009097 | *SLC35F3* | No |
| 100 | chr1 | q42.2 | 234 306 710 | 234 306 769 | 1 | 0 | 0 | -0,48579 | 0 | 0,01257 | *SLC35F3* | No |
| 103 | chr1 | q42.2 | 234 624 182 | 234 624 241 | 1 | 0 | 0,306346 | 0 | 0 | 1,375 |  | No |
| 104 | chr1 | q42.2 | 234 687 892 | 234 687 951 | 1 | 0 | 0 | -0,33477 | 0 | 0,3227 |  | No |
| 105 | chr1 | q42.3 | 234 843 707 | 234 843 766 | 1 | 0 | 0 | -0,32024 | 0 | 0,4951 |  | No |
| 107 | chr7 | p14.3 | 31 137 964 | 31 138 023 | 1 | 0 | 0 | -0,36906 | 0 | 0,2489 | *ADCYAP1R1* | No |
| 108 | chr7 | p14.3 | 31 362 446 | 31 362 505 | 1 | 0 | 0,319445 | 0 | 0 | 0,4282 |  | No |
| 109 | chr7 | p14.3 | 31 522 983 | 31 523 042 | 1 | 0 | 0 | -0,49643 | 0 | 0,01055 |  | No |
| 110 | chr7 | p14.3 | 31 708 933 | 31 708 992 | 1 | 0 | 0,320619 | 0 | 0 | 0,07699 |  | No |
| 111 | chr7 | p14.3 | 31 826 301 | 31 826 360 | 1 | 0 | 0,423233 | 0 | 0 | 0,0253 | *PDE1C* | No |
| 114 | chr7 | p14.3 | 32 241 823 | 32 241 882 | 1 | 0 | 0 | -0,3386 | 0 | 0,07116 | *PDE1C* | No |
| 115 | chr7 | p14.3 | 32 291 770 | 32 298 007 | 2 | 0 | 0 | -0,3077 | 0 | 0,02129 | *PDE1C* | No |
| 117 | chr7 | p14.3 | 32 307 749 | 32 307 808 | 1 | 0 | 0,465084 | 0 | 0 | 0,01558 | *PDE1C* | No |
| 118 | chr7 | p14.3 | 32 343 141 | 32 343 200 | 1 | 0 | 0 | -0,37313 | 0 | 0,04798 |  | No |
| 119 | chr7 | p14.3 | 32 497 156 | 32 497 215 | 1 | 0 | 0 | -0,45056 | 0 | 0,03394 |  | No |
| 122 | chr7 | p14.3 | 32 871 401 | 32 871 460 | 1 | 0 | 0 | -0,38249 | 0 | 0,04376 |  | No |
| 123 | chr7 | p14.3 | 32 998 408 | 32 998 452 | 1 | 0 | 0,364178 | 0 | 0 | 0,1405 | *FKBP9* | No |
| 125 | chr7 | p14.3 | 33 287 393 | 33 287 452 | 1 | 0 | 0 | -0,33957 | 0 | 0,06519 | *BBS9* | No |
| 126 | chr7 | p14.3 | 33 367 924 | 33 367 983 | 1 | 0 | 0,331571 | 0 | 0 | 0,06891 | *BBS9* | No |
| 127 | chr7 | p14.3 | 33 517 589 | 33 517 648 | 1 | 0 | 0,312426 | 0 | 0 | 0,08484 | *BBS9* | No |
| 129 | chr7 | p14.3 | 33 576 817 | 33 576 875 | 1 | 0 | 0,333901 | 0 | 0 | 0,7981 | *BBS9* | No |
| 130 | chr7 | p14.3 | 33 631 682 | 33 631 741 | 1 | 0 | 0 | -0,35934 | 0 | 0,05137 | *BBS9* | No |
| 131 | chr7 | p14.3 | 33 860 404 | 33 860 463 | 1 | 0 | 0 | -0,31464 | 0 | 0,04136 |  | No |
| 132 | chr7 | p14.3 | 33 892 972 | 33 893 031 | 1 | 0 | 0,471398 | 0 | 0 | 0,03097 |  | No |
| 133 | chr7 | p14.3 | 34 019 727 | 34 019 786 | 1 | 0 | 0 | -0,30983 | 0 | 0,04418 | *BMPER* | No |
| 134 | chr7 | p14.3 | 34 049 396 | 34 049 455 | 1 | 0 | 0,329316 | 0 | 0 | 0,6537 | *BMPER* | No |
| 136 | chr7 | p14.3 | 34 334 095 | 34 334 154 | 1 | 0 | 0,316521 | 0 | 0 | 0,1626 |  | No |
| 137 | chr7 | p14.3 | 34 590 655 | 34 590 714 | 1 | 0 | 0 | -0,48677 | 0 | 0,01236 | *AAA1* | No |
| 138 | chr7 | p14.3 | 34 771 949 | 34 772 008 | 1 | 0 | 0 | -0,57788 | 0 | 0,00535 | *AAA1, NPSR1* | No |
| 1 | chr1 | p21.3 | 95 153 061 | 95 153 120 | 1 | 0 | 0 | -0,33043 | 0 | 0,07421 |  | Yes |
| 4 | chr1 | p21.3 | 95 412 956 | 95 413 015 | 1 | 0 | 0 | -0,35686 | 0 | 0,0542 | *LOC729970* | Yes |
| 6 | chr1 | p21.3 | 95 928 948 | 95 929 007 | 1 | 0 | 0,305649 | 0 | 0 | 0,7545 |  | Yes |
| 9 | chr1 | p21.3 | 97 203 547 | 97 203 606 | 1 | 0 | 0 | -0,41172 | 0 | 0,0321 | *PTBP2* | Yes |
| 11 | chr1 | p21.3 | 97 344 942 | 97 345 001 | 1 | 0 | 0 | -0,44964 | 0 | 0,01907 |  | Yes |
| 15 | chr1 | p21.3 | 97 972 784 | 97 972 843 | 1 | 0 | 0 | -0,57379 | 0 | 0,004104 | *DPYD* | Yes |
| 16 | chr1 | p21.3 | 98 010 478 | 98 010 537 | 1 | 0 | 0 | -0,3851 | 0 | 0,03944 | *DPYD* | Yes |
| 19 | chr1 | p21.3 | 98 234 234 | 98 234 293 | 1 | 0 | 0 | -0,38771 | 0 | 0,03989 | *DPYD* | Yes |
| 23 | chr1 | p21.3 | 99 138 542 | 99 138 601 | 1 | 0 | 0 | -0,33027 | 0 | 0,07265 | *SNX7* | Yes |
| 24 | chr1 | p21.3 | 99 230 386 | 99 230 445 | 1 | 0 | 0 | -0,37146 | 0 | 0,04605 |  | Yes |
| 25 | chr1 | p21.3 | 99 258 087 | 99 258 146 | 1 | 0 | 0 | -0,31331 | 0 | 0,08434 |  | Yes |
| 26 | chr1 | p21.3 | 99 407 187 | 99 407 246 | 1 | 0 | 0 | 0 | -1,17675 | 2,17E-07 | *LPPR5* | Yes |
| 27 | chr1 | p21.3 | 99 530 526 | 99 530 585 | 1 | 0 | 0,347624 | 0 | 0 | 0,191 | *LOC100129620* | Yes |
| 30 | chr1 | p21.2 | 100 155 279 | 100 155 338 | 1 | 0 | 0 | -0,47035 | 0 | 0,3078 | *PALMD, MIR548D1, MIR548AA1* | Yes |
| 33 | chr1 | p21.2 | 100 296 451 | 100 296 510 | 1 | 0 | 0 | -0,37394 | 0 | 0,04441 |  | Yes |
| 34 | chr1 | p21.2 | 100 393 488 | 100 393 547 | 1 | 0 | 0 | -0,7658 | 0 | 0,000735 |  | Yes |
| 36 | chr1 | p21.2 | 100 464 888 | 100 464 947 | 1 | 0 | 0 | -0,34856 | 0 | 0,05866 | *SLC35A3* | Yes |
| 37 | chr1 | p21.2 | 100 614 291 | 100 614 350 | 1 | 0 | 0 | -0,35019 | 0 | 0,06499 | *CCDC76* | Yes |
| 50 | chr1 | p21.1 | 102 813 875 | 102 813 934 | 1 | 0 | 0 | -0,3851 | 0 | 0,03918 |  | Yes |
| 54 | chr1 | p21.1 | 103 418 213 | 103 418 272 | 1 | 0 | 0 | -0,33412 | 0 | 0,8703 | *COL11A1* | Yes |
| 55 | chr1 | p21.1 | 103 520 963 | 103 521 022 | 1 | 0 | 0 | -0,63201 | 0 | 0,005997 | *COL11A1* | Yes |
| 57 | chr1 | p21.1 | 104 210 997 | 104 211 056 | 1 | 0 | 0,307473 | 0 | 0 | 0,08831 |  | Yes |
| 63 | chr1 | p21.1 | 104 665 809 | 104 665 868 | 1 | 0 | 0 | -0,57998 | 0 | 0,003431 |  | Yes |
| 64 | chr1 | p21.1 | 104 984 792 | 104 984 851 | 1 | 0 | 0 | -0,41967 | 0 | 0,02911 |  | Yes |
| 67 | chr1 | p21.1 | 106 021 887 | 106 021 946 | 1 | 0 | 0,720453 | 0 | 0 | 0,000718 |  | Yes |
| 68 | chr1 | p21.1 | 106 099 025 | 106 099 084 | 1 | 0 | 0 | -0,47423 | 0 | 0,01542 |  | Yes |
| 69 | chr1 | p21.1 | 106 129 489 | 106 129 548 | 1 | 0 | 0 | 0 | -1,32863 | 5,64E-08 |  | Yes |
| 71 | chr1 | p13.3 | 107 236 027 | 107 236 086 | 1 | 0 | 0 | -0,43245 | 0 | 0,02239 |  | Yes |
| 77 | chr1 | p13.3 | 108 404 168 | 108 404 227 | 1 | 0 | 0 | -0,32507 | 0 | 0,0741 | *VAV3* | Yes |
| 81 | chr1 | p13.3 | 108 789 087 | 108 789 146 | 1 | 0 | 0,336229 | 0 | 0 | 0,9947 |  | Yes |
| 83 | chr1 | p13.3 | 108 926 313 | 108 926 372 | 1 | 0 | 0,527011 | 0 | 0 | 0,421 |  | Yes |
| 82 | chr1 | p13.3 | 108 926 313 | 108 968 593 | 2 | 0 | 0,373772 | 0 | 0 | 0,006213 |  | Yes |
| 86 | chr1 | p13.3 | 109 460 719 | 109 460 778 | 1 | 0 | 0 | -0,36126 | 0 | 0,05064 | *GPSM2* | Yes |
| 90 | chr1 | p13.3 | 109 982 205 | 109 982 264 | 1 | 0 | 0,374773 | 0 | 0 | 1,894 |  | Yes |
| 89 | chr1 | p13.3 | 109 982 205 | 109 987 611 | 2 | 0 | 0,332011 | 0 | 0 | 0,01344 |  | Yes |
| 91 | chr1 | q42.2 | 233 220 560 | 233 220 619 | 1 | 0 | 0 | -0,34017 | 0 | 0,7805 | *PCNXL2* | Yes |
| 93 | chr1 | q42.2 | 233 287 984 | 233 288 043 | 1 | 0 | 0 | -0,35254 | 0 | 0,05891 | *PCNXL2* | Yes |
| 95 | chr1 | q42.2 | 233 708 670 | 233 708 729 | 1 | 0 | 0,464008 | 0 | 0 | 0,01533 |  | Yes |
| 99 | chr1 | q42.2 | 234 257 957 | 234 258 016 | 1 | 0 | 0 | -0,36155 | 0 | 0,05399 | *SLC35F3* | Yes |
| 101 | chr1 | q42.2 | 234 528 171 | 234 528 228 | 1 | 0 | 0 | -0,56618 | 0 | 0,004269 | *TARBP1* | Yes |
| 102 | chr1 | q42.2 | 234 603 293 | 234 603 352 | 1 | 0 | 0 | -0,33707 | 0 | 0,0689 | *TARBP1* | Yes |
| 106 | chr7 | p14.3 | 31 002 780 | 31 005 134 | 2 | 0 | 0,322635 | 0 | 0 | 0,123 | *GHRHR* | Yes |
| 112 | chr7 | p14.3 | 31 848 664 | 31 848 723 | 1 | 0 | 0 | -0,34025 | 0 | 0,9157 | *PDE1C* | Yes |
| 113 | chr7 | p14.3 | 32 062 882 | 32 062 941 | 1 | 0 | 0 | -0,42151 | 0 | 0,02607 | *PDE1C* | Yes |
| 116 | chr7 | p14.3 | 32 297 949 | 32 298 007 | 1 | 0 | 0 | -0,3481 | 0 | 2,028 | *PDE1C* | Yes |
| 120 | chr7 | p14.3 | 32 552 237 | 32 552 283 | 1 | 0 | 0,47731 | 0 | 0 | 0,1656 | *AVL9* | Yes |
| 121 | chr7 | p14.3 | 32 573 006 | 32 573 065 | 1 | 0 | 0 | -0,39937 | 0 | 0,03717 | *AVL9* | Yes |
| 124 | chr7 | p14.3 | 33 111 959 | 33 112 018 | 1 | 0 | 0 | -0,3027 | 0 | 0,236 |  | Yes |
| 128 | chr7 | p14.3 | 33 551 428 | 33 551 487 | 1 | 0 | 0 | -0,3548 | 0 | 0,05452 | *BBS9* | Yes |
| 135 | chr7 | p14.3 | 34 153 606 | 34 153 665 | 1 | 0 | 0 | -0,35386 | 0 | 1,007 | *BMPER* | Yes |
| 139 | chr7 | p14.3 | 34 968 085 | 34 968 144 | 1 | 0 | 0 | -0,44322 | 0 | 0,02913 |  | Yes |
| 140 | chr7 | p14.3 | 34 991 038 | 34 991 097 | 1 | 0 | 0 | -0,47689 | 0 | 0,01432 | *DPY19L1* | Yes |
