# Supplementary material for: Familial Infertility (Azoospermia and Cryptozoospermia) in Two Brothers—Carriers of t(1;7) Complex Chromosomal Rearrangement (CCR): Molecular Cytogenetic Analysis
Source: Int J Mol Sci. 2020 Jun 26;21(12):4559. doi: 10.3390/ijms21124559 (PMC7349667; doi:10.3390/ijms21124559)
Supplement: Supplementary file 1 [file ijms-21-04559-s001.zip › Supplementary Table6.docx]

**Supplementary Table S6** BAC clones used in breakpoint mapping of the chromosomes involved in CCR t(1;7). All BAC clones were also marked in Figure 1C.

| **chromosome arm/BAC clone** | ***locus*** | **position** | **result** |
| --- | --- | --- | --- |
| *1p* | | | |
| RP11-79I13 | 1p31.1 | 81,707,225-81,853,567 | translocated on der(7) 7p |
| RP11-93B8 | 1p22.1-1p21.3 | 94,463,124-94,630,298 | translocated on der(7) 7p |
| *1q* | | | |
| RP11-134F15 | 1q42.13 | 229,556,852-229,718,263 | normal position |
| RP11-98M5 | 1q42.2 | 232,528,838-232,710,404 | normal position |
| RP11-67L19 | 1q42.3 | 233,566,930-233,745,426 | normal position |
| RP11-151E10 | 1q42.3 | 234,256,411-234,401,109 | translocated on der(1) 1p |
| *7p* | | | |
| RP11-539B24 | 7p14.3 | 31,525,818-31,667,326 | translocated on der(1) 1q |
| RP11-46A17 | 7p14.3 | 34,388,646-34,570,764 | normal position |
| RP11-75O22 | 7p14.2 | 37,18-37,37 | normal position |
