# Supplementary figures and images for: Familial Infertility (Azoospermia and Cryptozoospermia) in Two Brothers—Carriers of t(1;7) Complex Chromosomal Rearrangement (CCR): Molecular Cytogenetic Analysis
Source: Int J Mol Sci. 2020 Jun 26;21(12):4559. doi: 10.3390/ijms21124559 (PMC7349667; doi:10.3390/ijms21124559)

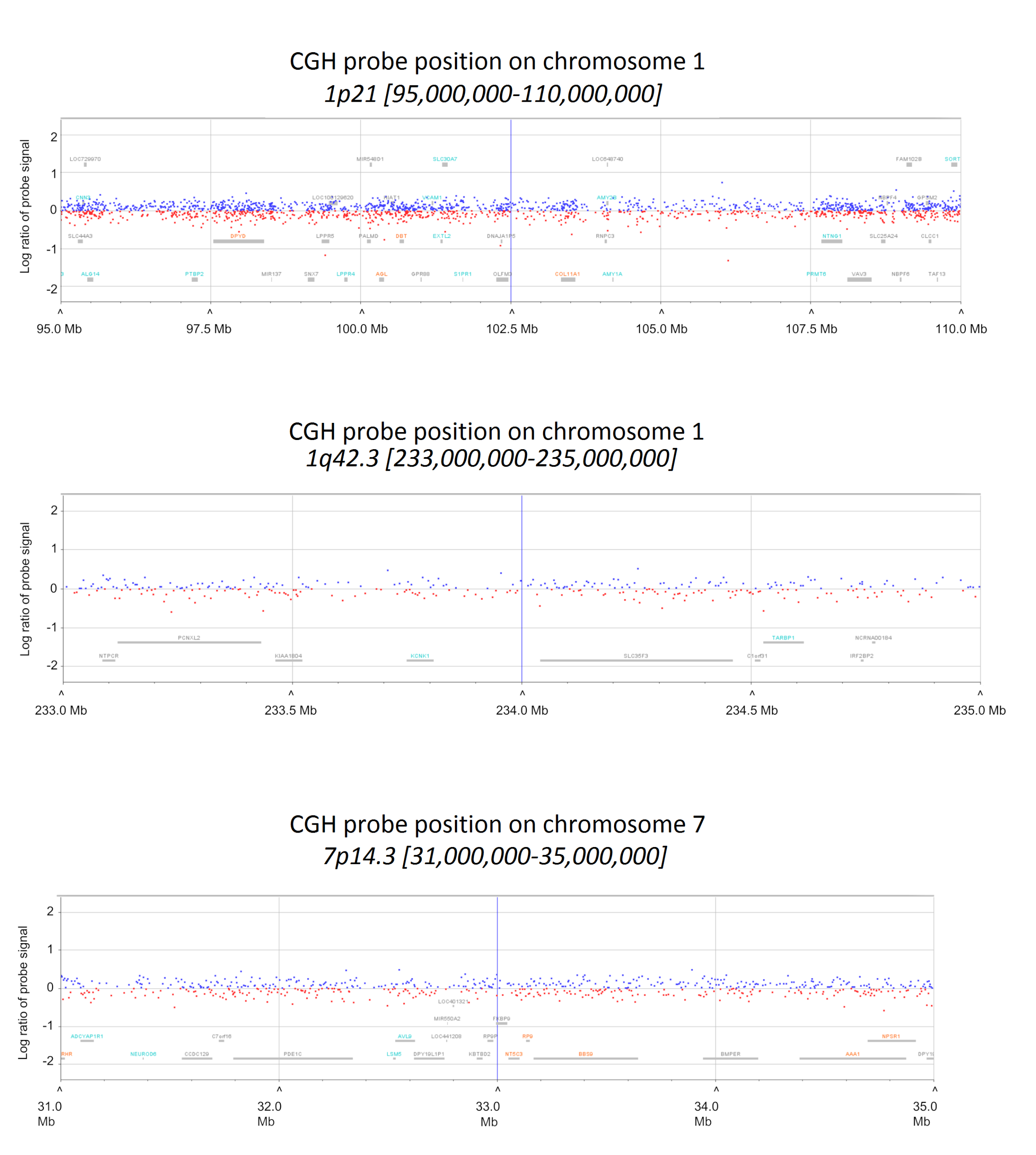

Supplement: Supplementary file 1 [file ijms-21-04559-s001.zip › Suppl Figure 1.tif]
